# Synthesis, Crystal Structure, and Supramolecular Understanding of 1,3,5-*Tris*(1-phenyl-1*H*-pyrazol-5-yl)benzenes

**DOI:** 10.3390/molecules23010022

**Published:** 2017-12-22

**Authors:** Marcos A. P. Martins, Alexandre R. Meyer, Paulo R. S. Salbego, Daniel M. dos Santos, Guilherme A. de Moraes, Helio G. Bonacorso, Nilo Zanatta, Clarissa P. Frizzo, Manfredo Hörner

**Affiliations:** 1 Núcleo de Química de Heterociclos (NUQUIMHE), Department of Chemistry, Federal University of Santa Maria (UFSM), 97105-900 Santa Maria, Brazil; alexandrerobisonmeyer@gmail.com (A.R.M.); paulosalbego@gmail.com (P.R.S.S.); santosmarquesdaniel@gmail.com (D.M.d.S.); helio.bonacorso@ufsm.br (H.G.B.); nilo.zanatta@ufsm.br (N.Z.); clarissa.frizzo@gmail.com (C.P.F.); 2 Núcleo de Investigação de Triazenos e Complexos (NITRICO), Department of Chemistry, Federal University of Santa Maria (UFSM), 97105-900 Santa Maria, Brazil; guilhermemoraes18@yahoo.com.br (G.A.d.M.); hoerner.manfredo@gmail.com (M.H.)

**Keywords:** pyrazoles, crystal structure, QTAIM, NMR, X-ray, supramolecular chemistry, crystal engineering

## Abstract

Understanding the supramolecular environment of crystal structures is necessary to facilitate designing molecules with desirable properties. A series of 12 novel 1,3,5-*tris*(1-phenyl-1*H*-pyrazol-5-yl)benzenes was used to assess the existence of planar stacking columns in supramolecular structures of pyrazoles. This class of molecules with different substituents may assist in understanding how small structural changes affect the supramolecular environment. The obtained compounds did not present the formation of planar stacking interactions between benzenes in solid or liquid states. This supposition was indicated by single crystal diffraction, Density Functional Theory (DFT) and quantum theory of atoms in molecules (QTAIM) calculations, and concentration-dependent liquid-state ^1^H nuclear magnetic resonance (NMR). NMR showed that chemical shifts of benzene and pyrazole hydrogens confirm that planar stacking interactions are not formed in solution. The crystalline structures presented different molecular conformations. The molecular structures of **5** and **9b** are in a twisted conformation, while compound **7** showed a conformation analogous to a calyx form.

## 1. Introduction

When designing new molecules with enhanced properties, analysis should go beyond merely investigating the molecular structure [1,2]. It is necessary to understand the supramolecular environment, especially in the self-assembly of the desired molecules [3,4].

Heterocycles, and especially pyrazoles, may present supramolecular structures with columnar stacked systems, such as one-dimensional constructs. As a result, these molecules present potentially enhanced luminescent and fluorescent properties [5,6]. However, different supramolecular arrangements can be observed between structures with similar molecular structures, which explains why small structural differences can drastically affect the self-assembly observed both in solution and in solid states [2,7].

**Figure 1:**
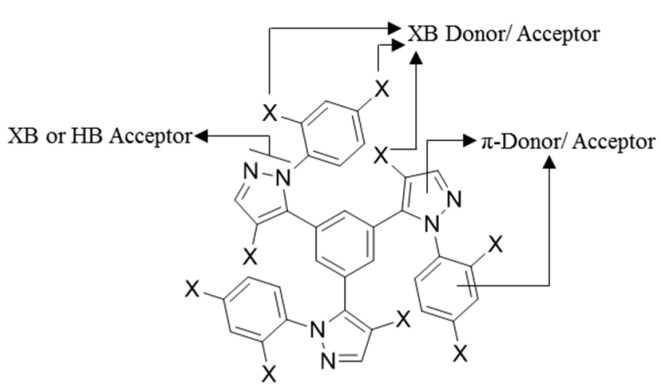
Intermolecular interaction sites of 1,3,5-*tris*(1-phenyl-1*H*-pyrazol-5-yl)benzenes.

The 1,3,5-trispyrazolylbenzenes are flat, disk-shaped molecules that can potentially present as column formations in their self-assembly. However, different applications of these molecules may be observed depending on the formation of different supramolecular arrangements, including ligands for metal−organic framework (MOFs) formation [8]. In recent years, a series of pyrazoles with different molecular structures have been synthesized by our research group [9]. The synthesis of 1,3,5-*tris*(1-phenyl-1*H*-pyrazol-5-yl)benzenes (TPB) was developed presenting a complex molecular structure with chemical functionalities that may form a wide variety of intermolecular interactions (Figure 1).

This class of compounds is an excellent model to study the influence of subtle substituent modifications on supramolecular arrangement. Thus, the main goal of this study is to present novel TPB compounds, understand their self-assembly, and assess whether their stacking formation is possible. Therefore, single crystal X-ray diffraction, QTAIM calculations, and liquid state NMR experiments were performed.

## 2. Results and Discussion

### 2.1. Synthesis and Characterization

Enaminones are versatile reagents easily obtained and widely applied in heterocyclic synthesis [10]. They can be obtained by condensation reactions of carbonyl compounds with *N*,*N*-dimethylformamide diethyl acetal in refluxing aromatic hydrocarbons, ethanol, ether, toluene, DMF, or without solvents [10]. Microwave irradiation can also employed be in the synthesis of enaminones [11]. The enaminone 1,3,5-*tris*(3-dimethylamino-1-oxoprop-2-en-yl)benzene **1** was synthesized according to a synthetic methodology adapted from the literature [11,12,13].

The synthesis of pyrazoles, based on cyclocondensation between enaminones and hydrazines, is already well established in the literature. Several synthetic methodologies have been applied in the synthesis of alkyl and aryl-1*H*-pyrazoles, including reflux in ethanol, methanol, propanol, acetic acid, microwave irradiation, and ionic liquid [14,15,16,17,18,19,20,21].

**Scheme 1:**
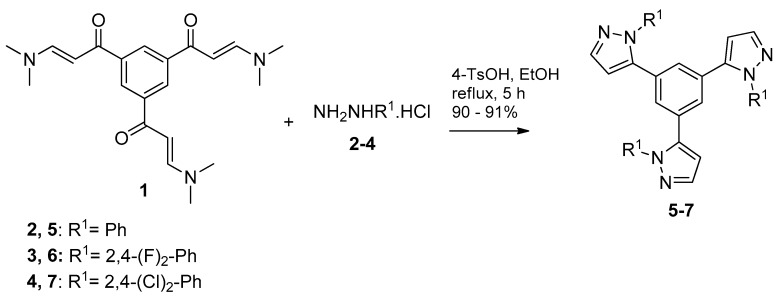
Synthesis of 1,3,5-*tris*(1-phenyl-1*H*-pyrazol-5-yl)benzenes **5**–**7**.

**Figure 2:**
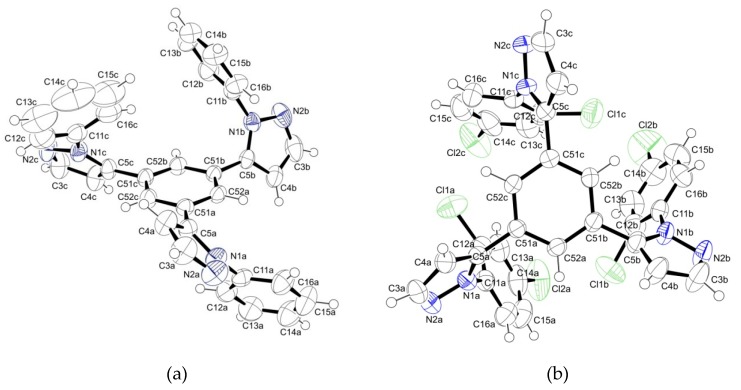
ORTEP diagrams of 1,3,5-*tris*(1-phenyl-1*H*-pyrazol-5-yl)benzenes **5** (**a**) and **7** (**b**) drawn as 50% ellipsoids.

The 1,3,5-*tris*(1-phenyl-1*H*-pyrazol-5-yl)benzenes **5**–**7** were obtained by a cyclization reaction of enaminone **1** with phenylhydrazines **2**–**4** catalyzed by 4-toluenesulfonic acid in ethanol reflux (Scheme 1). The product structures were confirmed by spectroscopic data (See Supplementary Materials, Figures S3–S8 and S27–S29). Its ^1^H-NMR spectra show that the signal of the benzene hydrogens appears in singlet form with chemical shifts in the range of 6.92–6.99 ppm. This signal indicates that the three substituents at positions 1, 3, and 5 of the benzene are equal. Formation of the pyrazole ring is confirmed by the presence of two doublets with coupling constant ^3^*J* = 2 Hz and chemical shifts in the ranges of 6.13–6.20 and 7.43–7.64 ppm. The formation of the *1,5*-isomer for compounds **5** and **7** was confirmed by X-ray diffraction (Figure 2 and Table S1).

**Scheme 2:**
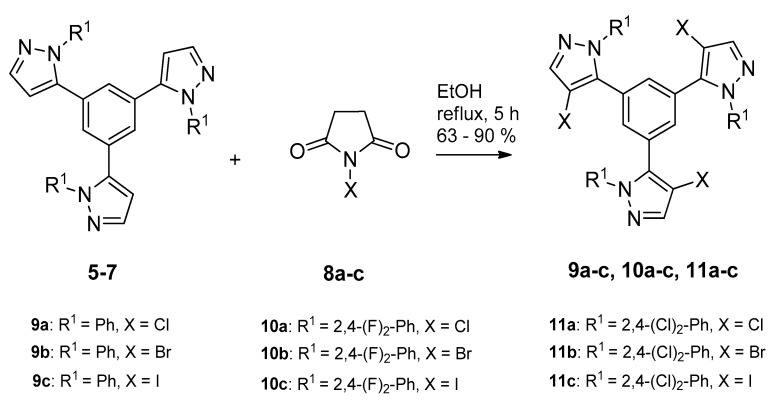
Synthesis of 1,3,5-*tris*(1-phenyl-4-halo-1*H*-pyrazol-5-yl)benzenes **9**–**11**.

**Figure 3:**
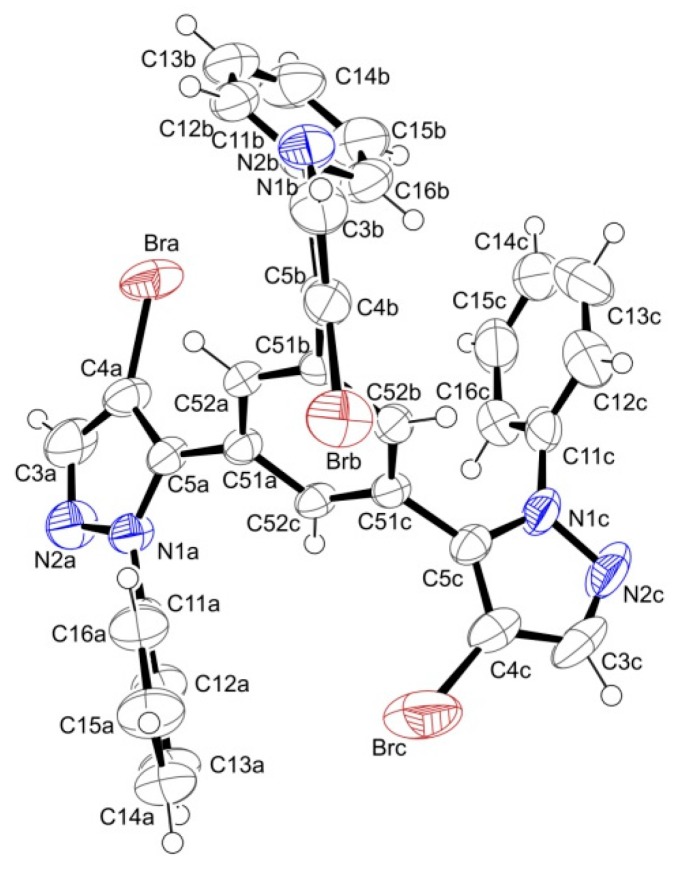
ORTEP diagram of 1,3,5-*tris*(1-phenyl-4-halo-1*H*-pyrazol-5-yl)benzene **9b** drawn as 50% ellipsoids.

Several synthetic methodologies have been employed in halogenation reactions of pyrazoles, including: CCl_4_, ethanol, water, DMF, acetonitrile, dichloromethane, THF, and ethyl acetate [22,23,24,25,26,27,28,29,30,31]. The halogenation reactions of the 1,3,5-*tris*(1-phenyl-1*H*-pyrazol-5-yl)benzenes **5**–**7** were performed with *N*-halosuccinimides **8a**–**c** in ethanol reflux (Scheme 2). The structures of products **9**–**11** were confirmed by spectroscopic data (See Supplementary Materials, Figures S9–S26 and S30–S38). In its ^1^H-NMR spectra, the signal referent to the hydrogen of position 4 of the pyrazole ring was not observed, while the hydrogen present at position 3 was observed in singlet form in the chemical shift range of 7.64–7.78 ppm. The chemical shift of the carbon signal at position 4 of the pyrazole ring in the ^13^C-NMR spectra changes from the range of 106.9–108.0 ppm to 110.8–111.6 ppm, 95.8–96.6 ppm, and 62.0–63.4 ppm, respectively, for the chlorinated, brominated, and iodinated pyrazoles. Halogenation at position 4 of the pyrazole was also confirmed by X-ray diffraction of compound **9b** (Figure 3 and Table S1). At this moment, suitable single crystals for X-ray diffraction were obtained solely for three compounds (**5**, **7**, and **9b**).

### 2.2. Supramolecular Arrangement 

**Figure 4:**
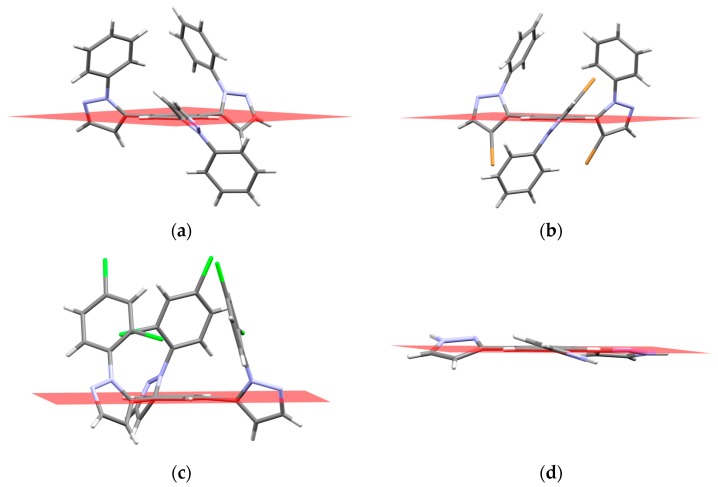
Molecular conformation of compounds **5** (**a**) and **9b** (**b**) in twisted form; compound **7** (**c**) in calyx form; and compound LIJJIY (CSD refcode) [6] in planar conformation (**d**).

The crystalline structures for compounds **5**, **7**, and **9b** presented with different molecular conformations (Figure 4). Compounds **5** and **9b** presented molecular structures with a twisted conformation, and compound **7** showed a distinct conformation analogous to a calyx form. In this primary analysis, the crystalline structures presented a deviation from the benzene plane. In compounds **5** and **9b**, one of the phenyl-1*H*-pyrazole-5-yl groups is in one direction of the benzene plane (e.g., under the traced plane) while the other two groups are in the opposite direction (e.g., above the plane) (Figure 4a,b). The calyx form of compound **7** showed all phenyl-1*H*-pyrazole-5-yl group portions in the same direction (Figure 4c).

**Figure 5:**
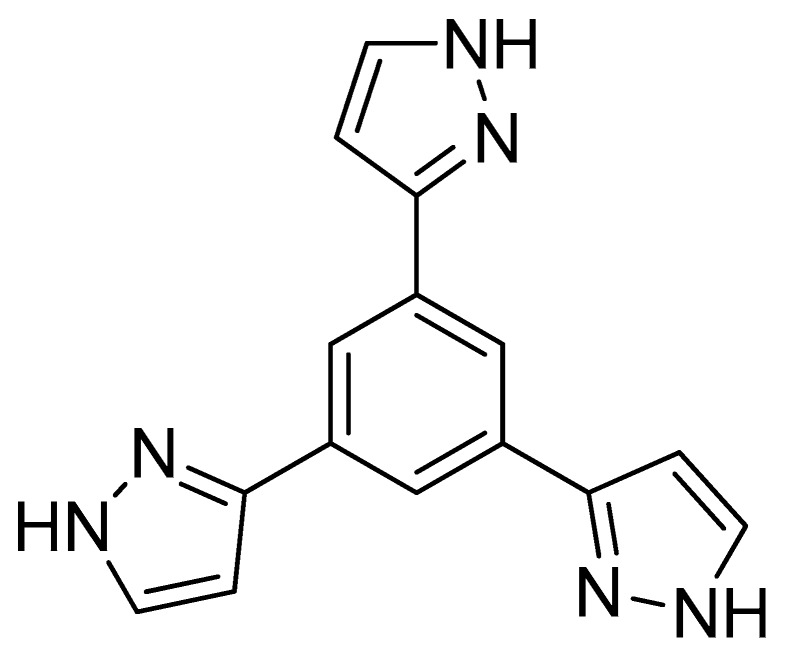
Molecular structure of 1,3,5-tris(1*H*-pyrazol-3-yl)benzene (refcode LIJJIY).

Only one example of a similar structure was found in the Cambridge Structure Database. Zhao et al. [6] reported a 1,3,5-tris(1*H*-pyrazol-3-yl)benzene (Figure 5) (refcode LIJJIY) presenting with a planar conformation, showing all three phenyl-1*H*-pyrazole-3-yl groups in almost the same plane of the benzene ring (Figure 4d).

**Figure 6:**
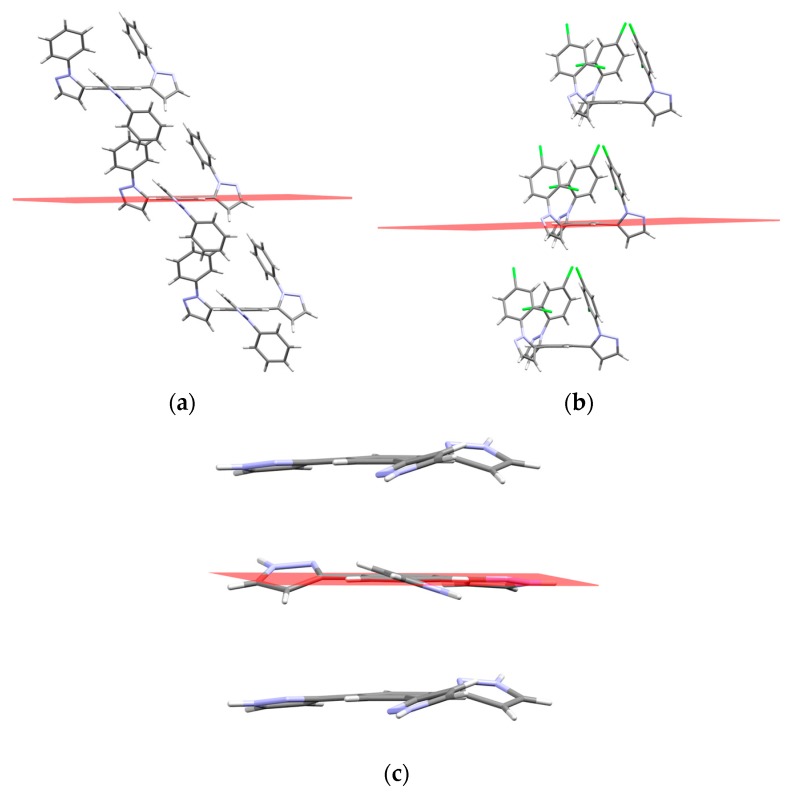
1D arrangement for compounds **5** (**a**), **7** (**b**), and LIJJIY [6] (**c**).

This initial assessment indicates a difficulty in the columnar packing formation of these structures, although further investigation of crystal packing of these molecules should be performed. Therefore, the possible 1D motif formed in these structures was investigated in order to assess the kind of packing present in each one. Then, the observed packing was compared with the model reported by Zhao et al., which shows planar 1D column formation (Figure 6c). Since compounds **5** and **9b** showed the same molecular behavior and consequently the same 1D supramolecular arrangement, only **5** was showed. The 1D arrangement for **9b** can be observed in the Supplementary Materials (Figure S1).

The 1D projection for the structures, and differences when compared with the structure reported by Zhao et al. [6], can be seen in Figure 6. In our case, planar stacking, which is a parallel interaction between the benzene rings of structures, was not observed. The observed columns occur in a different form because of the conformational characteristics of each molecule. In compound **5**, QTAIM analysis revealed one C-H∙∙∙π and six H∙∙∙H intermolecular interactions in each dimer of the 1D arrangement. The C-H∙∙∙π interaction occurs between a hydrogen atom of one of the phenyl rings and a carbon atom from the benzene ring. This is the second highest value of ρ (electronic density in the critical point), which indicates the magnitude of the interaction strength (See Supplementary Materials, Figure S2 and Table S4). Moreover, the dimers involved in the 1D arrangement of compound **7** presented one π···π and two C-H∙∙∙Cl interactions. The π···π interaction occurs involving the carbon atom of one of the phenyl rings and a carbon atom from the central benzene ring, showing the highest value of ρ with 55% of the total dimer value (See Supplementary Materials, Figure S2 and Table S4). The two dimers observed in the arrangement of compound **9b** showed different interactions: one with seven interactions (four C-H∙∙∙N, one π∙∙∙π, and two C∙∙∙H) and the other with four C-H∙∙∙Br interactions. The first dimer showed low ρ values for the C-H∙∙∙N interactions, presenting the highest values for the C∙∙∙H and π∙∙∙π interactions (See Supplementary Materials, Figure S2 and Table S4).

This kind of approach can provide information regarding the possibility of stacking interactions and guide the design of new molecules with desired properties. The structures obtained do not present planar 1D stacking motifs, which indicate they are not viable for fluorescent or luminescent methods, although they are desirable models for use as ligands in the formation of metallic complexes. One remaining question regarding the self-assembly of these molecules is if the solid state behavior (crystal) is maintained in the liquid state. To answer this pertinent question, concentration-dependent liquid-state ^1^H NMR experiments were carried out for pyrazole **7** to evaluate the self-assembly of this compound.

**Figure 7:**
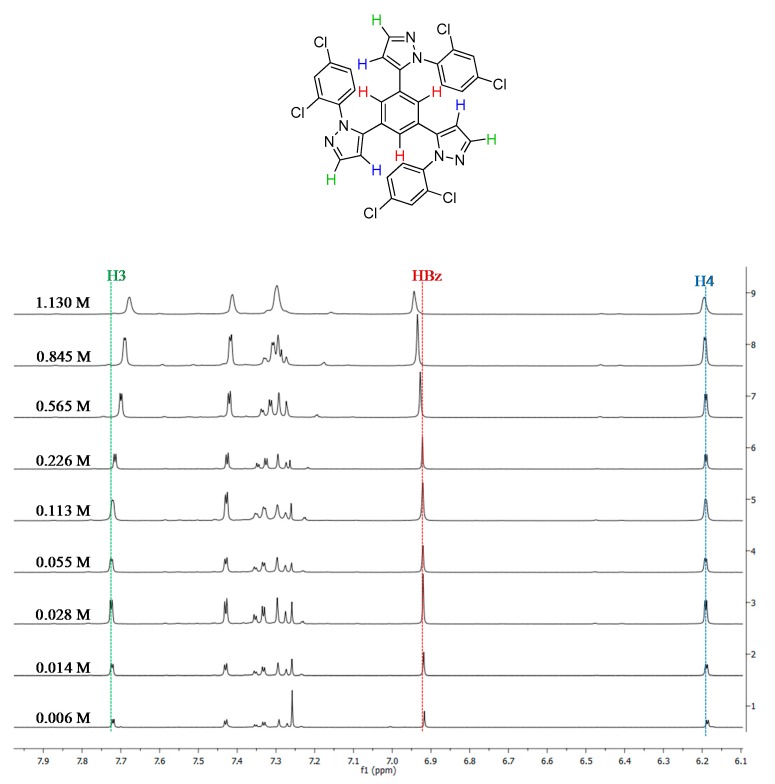
Concentration-dependent ^1^H NMR spectra of 1,3,5-*tris*(1-(2,4-dichlorophenyl)-1*H*-pyrazol-5-yl)benzene **7** performed in CDCl_3_ at 298 K.

The chemical shift changes of the hydrogens of the central benzene and pyrazoles were monitored. These nuclei are in a central position in the molecule and are ideal for evaluating the formation of stacking interactions. The data from the ^1^H NMR experiments (Figure 7) shows that the signal of the benzene hydrogens is deshielded according to increases in concentration. This behavior reveals that these hydrogens are involved in hydrogen bonds. The chemical shift of hydrogen H4 (in blue; Figure 7) of the pyrazole ring practically does not change. The most external hydrogen, H3 (in green; Figure 7), shows an upfield change in chemical shift. Therefore, the chemical shift changes of the benzene hydrogens and H4 hydrogen of the pyrazoles confirm that stacking interactions are not formed in solution.

### 2.3. QTAIM Analysis

Quantum Theory of Atoms in Molecules (QTAIM) [32,33] analysis was carried out to identify and assess the strength of the intramolecular interactions involved in the stabilization of the reported structures. Bond critical points (BCPs) were obtained to confirm existence of the interactions. QTAIM data on intramolecular interactions, which show BCPs, type of interaction, ρ, and E_INT_ are summarized in Table 1. Additional information on QTAIM data are available in the Supplementary Materials (Table S2). ρ and E_INT_ provide the magnitude of the interaction strength, since they represent the electronic density at the critical point and intramolecular interaction energy (obtained by E_INT_ = V/2) [34], respectively. V/2 can be used as an estimation of the intramolecular interaction strength [34].

The twisted structures **5** and **9b** present three and four intramolecular interactions, respectively. Compound **5** shows only C-H∙∙∙π interactions, where two are between the two phenyl groups (−0.22 and −0.33 kcal mol^−1^) and one is between the C-H from one phenyl group and the π-system from the central benzene ring with the highest contribution (−1.49 kcal mol^−1^). The 1,3,5-*tris*(1*H*-pyrazol-3-yl)benzene group above the benzene plane showed no interaction with another portion of the molecule, which may indicate the influence of intermolecular interactions on the molecular conformation. Compound **9b** presents two C-H∙∙∙π and two Br··· π interactions with almost the same magnitude, ranging from −0.35 to −0.61 kcal mol^−1^.

Compound **7** with the calyx-type molecular structure is stabilized by three major Cl∙∙∙π interactions (between −0.89 and −1.08 kcal mol^−1^) between the chlorine atom at position 2 from the phenyl group and the π-system from another phenyl ring. Additionally, a C-H∙∙∙Cl interaction was observed with a lower contribution to the stabilization energy, which only presented −0.44 kcal mol^−1^. Geometric data of the observed intramolecular interactions are presented in the Supplementary Materials (Table S3).

### 2.4. Molecular Elestrostatic Potential of Compounds ***5***, ***7***, and ***9b***

**Figure 8:**
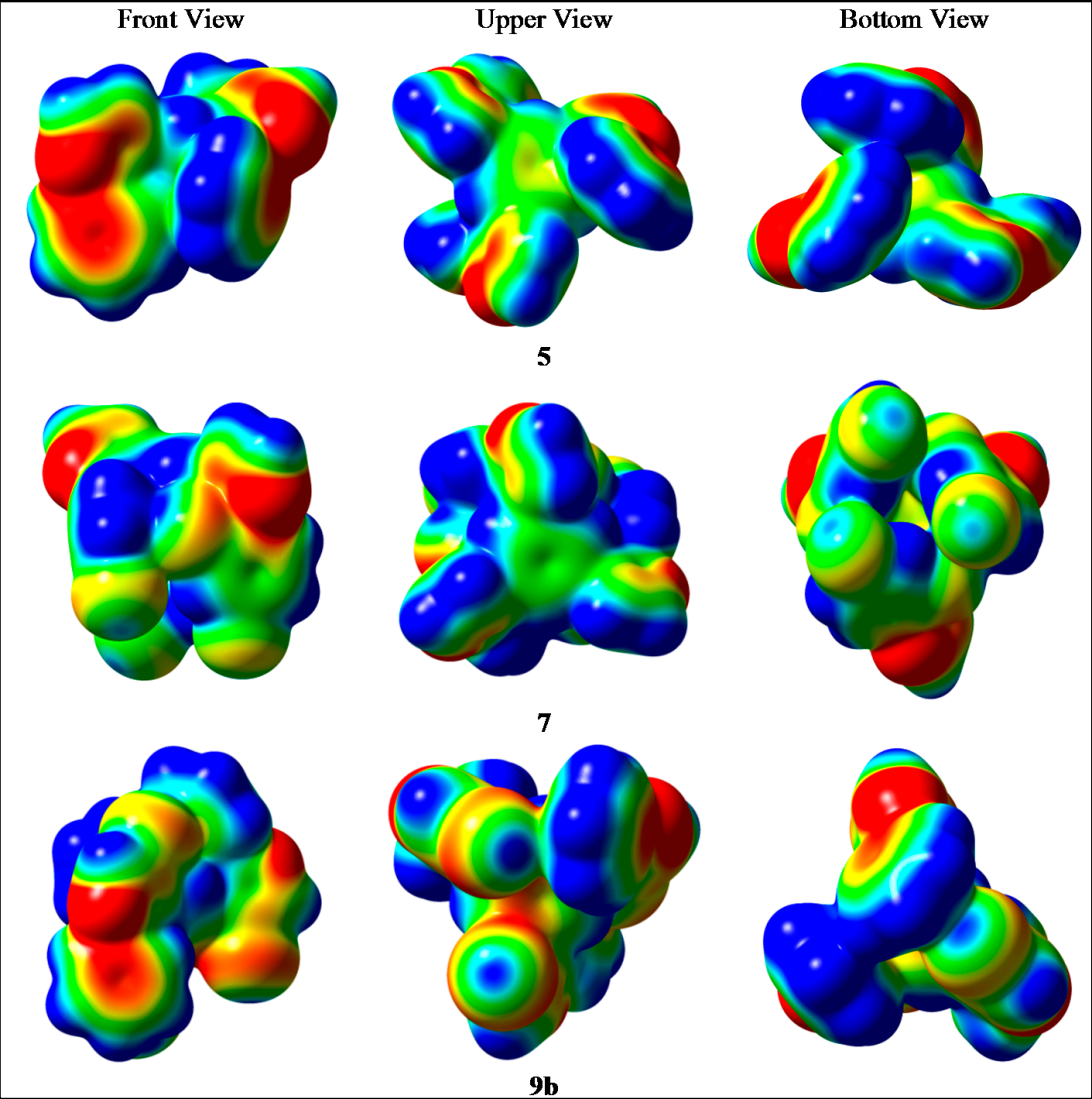
Molecular electrostatic potential of compounds **5**, **7**, and **9b**. Isodensity value of 0.001 at the ωB97X-D/cc-pVDZ level of theory with the aid of GaussView software. Red −0.01 au; Yellow −0.005 au; Green 0.00 and Blue 0.01 au.

The molecular electrostatic potential (MEP) [35,36] is a powerful tool to predict and understand noncovalent interactions [37,38,39,40,41,42]. The MEPs of **5**, **7,** and **9b** (Figure 8) were generated with the aim of analyzing the interaction sites of the molecules, leading to complementation of the QTAIM analysis. The MEP of compound **5** shows that the hydrogen atoms present positive electrostatic potentials, while the nitrogen atoms and π-system of the phenyl ring show negative electrostatic potentials. These opposite and complementary electrostatic potentials allied to its proximity in space allow the formation of the C-H∙∙∙π interactions observed in the molecular structure of **5** in the QTAIM analysis. The same behavior is observed for compound **9b**, although the presence of the Br∙∙∙π interaction can be also observed. This interaction occurs by the attraction of the negative electrostatic potential of the phenyl π-system by the positive electrostatic potential (σ-hole) localized on the outer side of the bromine atom. Compound **7** presents a distinct behavior from the other two compounds. In **7**, the chlorine atoms, which are attached to positions 2 and 4 of the phenyl group, showed negative electrostatic potentials in the equatorial region and small positive electrostatic potentials on the outer side of the atom. The electron withdrawing effect of the chlorine atoms turns the electrostatic potential of the phenyl ring slightly positive. Therefore, the region of negative electrostatic potential of the chlorine atom (position 2 of the phenyl) interacts with the phenyl ring and therefore causes Cl∙∙∙π interactions, resulting in the calyx form of compound **7**. 

## 3. Experimental Section

### 3.1. General Information

All reagents were purchased from commercial suppliers and used without further purification. The reactions under microwave irradiation were performed in a Discovery CEM MW using the mode operation without simultaneous cooling. The power of the equipment was established at 200 W, and a maximum level of internal pressure of 250 psi was used. ^1^H- and ^13^C-NMR spectra were recorded in a Bruker DPX-400 (^1^H at 400 MHz and ^13^C at 100 MHz) in CDCl_3_/TMS solutions at 298 K. Liquid chromatography coupled with tandem mass spectrometry (LC-MS/MS) was performed in an Agilent Model 6460 system with an electrospray ionization (ESI) source coupled to a QQQ detector. The voltage of the capillary was maintained at 3500 V. The source temperature was 300 °C with a flow rate of 5 L min^−1^. The jetstream temperature was 250 °C with a flow rate of 11 L min^−1^. All samples were injected with an autosampler, 1 μL by volume. Data were acquired in scan mode in the 50–500 *m*/*z* range and the product ion mode (product ion) MS/MS. The melting points were measured using a Microquıímica MQAPF 301 apparatus.

### 3.2. Synthesis

#### 3.2.1. General Procedure for Preparation of 1,3,5-*tris*(3-dimethylamino-1-oxoprop-2-en-yl)benzene (**1**)

Compound **1** was obtained according to methodology adapted from the literature [11,12]. A mixture of propanone (10 mmol), *N*,*N*-dimethylformamide diethyl acetal (5 mmol), and boron trifluoride etherate (3 drops) was added into a 10 mL microwave vessel equipped with a standard cap (vessel commercially furnished by Discover CEM). Afterwards, the vessel was sealed and the sample irradiated for 5 min at 150 °C. The propanone excess was evaporated under vacuum (reduced pressure) providing (E)-4-(dimethylamino)but-3-en-2-one. Acetic acid (20 mL) was added to the (E)-4-(dimethylamino)but-3-en-2-one (20 mmol) in a round-bottom flask equipped with a stir bar. The mixture was stirred at 118 °C in a pre-heated oil bath for 3 h. After cooling, 1,3,5-triacethylbenzene was separated from the reaction mixture by filtration and recrystallized in a 3:1 mixture of ethanol/dioxane. Finally, the 1,3,5-triacethylbenzene (1 mmol), *N*,*N*-dimethylformamide diethyl acetal (6 mmol), and boron trifluoride etherate (3 drops) were added to a microwave vessel equipped with a standard cap. The vessel was sealed and the sample irradiated in a microwave oven at 150 °C for 20 min. Compound **1** was obtained after the addition of 10 mL of *N*-hexane to the reaction mixture and subsequent filtration.

#### 3.2.2. General Procedure for Preparation of 1,3,5-*tris*(pirazolyl)benzenes **5**–**7**

The 1,3,5-*tris*-β-enaminone **1** (1 mmol), hydrochloride hydrazines **2**–**4** (3.6 mmol), and *p*-toluene sulfonic acid (0.6 mmol) were dissolved in dry ethanol (5 mL) in a round-bottom flask equipped with a stir bar. The reaction mixture was stirred at 78 °C in a pre-heated oil bath for 5 h. Afterwards, the ethanol was evaporated under reduced pressure and 1,3,5-*tris*(pirazolyl)benzenes were isolated with chloroform (10 mL) and washed with distillated water (3 × 10 mL). The organic phase was dried with anhydrous sodium sulfate, and the solvent evaporated under reduced pressure.

*1,3,5-Tris(1-phenyl-1H-pyrazol-5-yl)benzene* **5**. Yield 91%, m.p. 183–184 °C. ^1^H-NMR (400 MH_Z_, CDCl_3_, δ; ppm): 6.13 (d, ^3^*J *= 2, 3H, H4), 6.99 (s, 3H, HBz), 7.18–7.21 (m, 6H, HPh), 7.33–7.36 (m, 9H, HPh), 7.64 (d, ^3^*J* = 2, 3H, H3). ^13^C-NMR (100 MH_Z_, CDCl_3_, δ; ppm): 108.0 (C4), 125.5 (CAr), 128.0 (CAr), 128.7 (CAr), 129.1 (CAr), 131.3 (CAr), 139.8 (CAr), 140.4 (C3), 141.5 (C5). Positive ESI-MS *m*/*z* 505.2 [M + H]^+^.

*1,3,5-Tris(1-(2,4-difluorophenyl)-1H-pyrazol-5-yl)benzene* **6**. Yield 91%, m.p. 65–66 °C. ^1^H-NMR (400 MH_Z_, CDCl_3_, δ; ppm): 6.20 (d, ^3^*J *= 2, 3H, H4), 6.79–6.84 (m, 3H, 2-4-F-Ph), 6.95–7.00 (m, 3H, 2-4-F-Ph), 6.99 (s, 3H, HBz), 7.37–7.43 (m, 3H, 2-4-F-Ph), 7.64 (d, ^3^*J* = 2, 3H, H3). ^13^C-NMR (100 MH_Z_, CDCl_3_, δ; ppm): 107.3 (C4), 105.1 (dd, ^2^*J*_C–F_ = 26, ^2^*J*_C–F_ = 23, CAr), 112.2 (dd, ^2^*J*_C–F_ = 23, ^4^*J*_C–F_ = 4, CAr), 124.4 (dd, ^2^*J*_C–F_ = 12, ^4^*J*_C–F_ = 4, CAr) 127.4 (CAr), 129.9 (d, ^3^*J*_C–F_ = 10, CAr), 130.9 (CAr), 141.4 (C3), 143.4 (C5), 156.7 (dd, ^1^*J*_C–F_ = 256, ^3^*J*_C–F_ = 13, CAr), 162.6 (dd, ^1^*J*_C–F_ = 252, ^3^*J*_C–F_ = 11, CAr). Positive ESI-MS *m*/*z* 613.1 [M + H]^+^.

*1,3,5-Tris(1-(2,4-dichlorophenyl)-1H-pyrazol-5-yl)benzene* **7**. Yield 90%, m.p. 169–170 °C. ^1^H-NMR (400 MH_Z_, CDCl_3_, δ; ppm): 6.19 (d, ^3^*J *= 2, 3H, H4), 6.92 (s, 3H, HBz), 7.27 (s, 1H, 2-4-Cl-Ph), 7.30 (s, 2H, 2-4-Cl-Ph), 7.33 (d, ^3^*J *= 2, 2H, 2-4-Cl-Ph), 7.35 (d, ^3^*J *= 2, 1H, 2-4-Cl-Ph), 7.43 (d, ^3^*J *= 2, 3H, 2-4-Cl-Ph), 7.72 (d, ^3^*J *= 2, 3H, H3). ^13^C-NMR (100 MH_Z_, CDCl_3_, δ; ppm): 106.9 (C4), 127.4 (CAr), 128.0 (CAr), 130.3 (CAr), 130.6 (CAr), 130.8 (CAr), 133.0 (CAr), 135.9 (CAr), 136.4 (CAr), 141.3 (C3), 143.4 (C5). Positive ESI-MS *m*/*z* 710.8 [M + H]^+^.

#### 3.2.3. General Procedure for Preparation of 1,3,5-*Tris*(4-halo-pirazolyl)benzenes **9**–**11**

1,3,5-Tris(pirazolyl)benzenes **5**-**7** (0.25 mmol) and *N*-halosuccinimides **8** (2.25 mmol) were dissolved in ethanol (3 mL). The reactions of chlorination and bromination were performed in a round-bottom flask equipped with a stir bar in pre-heated oil bath for 5 h at 78 °C. For iodination, trifluoroacetic acid (0.25 mmol) was added and the reaction performed in a sealed tube, which was then heated in a pre-heated oil bath for 5 h at 78 °C. After cooling, pyrazole **9** was isolated from the reaction mixture by filtration and washed with dry ethanol (3 mL). For pyrazoles **10** and **11**, the ethanol was evaporated under reduced pressure, and the products isolated with chloroform (5 mL) and washed with distillated water (5 × 5 mL). The organic phase was dried with anhydrous sodium sulfate, and the solvent evaporated under reduced pressure.

*1,3,5-Tris(4-chloro-1-phenyl-1H-pyrazol-5-yl)benzene* **9a**. Yield 63%, m.p. 266–267 °C. ^1^H-NMR (400 MH_Z_, CDCl_3_, δ; ppm): 7.18 (s, 3H, HBz), 7.13–7.15 (m, 6H, HPh), 7.29–7.35 (m, 9H, HPh), 7.64 (s, 3H, H3). ^13^C-NMR (100 MH_Z_, CDCl_3_, δ; ppm): 111.6 (C4), 125.0 (CAr), 128.2 (CAr), 129.4 (CAr), 129.4 (CAr), 131.7 (CAr), 137.3 (C5), 139.7 (CAr), 139.1 (C3). Positive ESI-MS *m*/*z* 609.0 [M + H]^+^. 

*1,3,5-Tris(4-bromo-1-phenyl-1H-pyrazol-5-yl)benzene* **9b**. Yield 65%, m.p. 272–273 °C. ^1^H-NMR (400 MH_Z_, CDCl_3_, δ; ppm): 7.20 (s, 3H, HBz), 7.13–7.15 (m, 6H, HPh), 7.26–7.34 (m, 9H, HPh), 7.67 (s, 3H, H3). ^13^C-NMR (100 MH_Z_, CDCl_3_, δ; ppm): 96.6 (C4), 124.9 (CAr), 128.0 (CAr), 129.3 (CAr), 130.0 (CAr), 132.1 (CAr), 138.9 (C5), 139.6 (CAr), 141.0 (C3). Positive ESI-MS *m*/*z* 740.8 [M + H]^+^.

*1,3,5-Tris(4-iodo-1-phenyl-1H-pyrazol-5-yl)benzene* **9c**. Yield 66%, m.p. 299–300 °C. ^1^H-NMR (400 MH_Z_, CDCl_3_, δ; ppm): 7.16 (s, 3H, HBz), 7.13–7.15 (m, 6H, HPh), 7.24–7.31 (m, 9H, HPh), 7.72 (s, 3H, H3). ^13^C-NMR (100 MH_Z_, CDCl_3_, δ; ppm): 63.4 (C4), 124.8 (CAr), 128.0 (CAr), 129.3 (CAr), 131.4 (CAr), 132.9 (CAr), 139.5 (C5), 142.3 (CAr), 145.3 (C3). Positive ESI-MS *m*/*z* 662.8 [M + H]^+^.

*1,3,5-Tris(4-chloro-1-(2,4-difluorophenyl)-1H-pyrazol-5-yl)benzene* **10a**. Yield 85%, m.p. 195–196 °C. ^1^H-NMR (400 MH_Z_, CDCl_3_, δ; ppm): 6.76–6.81 (m, 3H, 2-4-F-Ph), 6.94–6.99 (m, 3H, 2-4-F-Ph), 7.18 (s, 3H, HBz), 7.39–7.45 (m, 3H, 2-4-F-Ph), 7.70 (s, 3H, H3). ^13^C-NMR (100 MH_Z_, CDCl_3_, δ; ppm): 105.3 (dd, ^2^*J*_C–F_ = 27, ^2^*J*_C–F_ = 24, CAr), 110.9 (C4), 112.5 (dd, ^2^*J*_C–F_ = 23, ^4^*J*_C–F_ = 4, CAr), 124.1 (dd, ^2^*J*_C–F_ = 12, ^4^*J*_C–F_ = 4, CAr) 128.5 (CAr), 129.7 (d, ^3^*J*_C–F_ = 10, CAr), 130.4 (CAr), 139.0 (C5), 140.1 (C3), 156.5 (dd, ^1^*J*_C–F_ = 256, ^3^*J*_C–F_ = 13, CAr), 162.9 (dd, ^1^*J*_C–F_ = 253, ^3^*J*_C–F_ = 11, CAr). Positive ESI-MS *m*/*z* 714.9 [M + H]^+^.

*1,3,5-Tris(4-bromo-1-(2,4-difluorophenyl)-1H-pyrazol-5-yl)benzene* **10b**. Yield 89%, m.p. 174–175 °C. ^1^H-NMR (400 MH_Z_, CDCl_3_, δ; ppm): 6.73–6.78 (m, 3H, 2-4-F-Ph), 6.90–6.95 (m, 3H, 2-4-F-Ph), 7.17 (s, 3H, HBz), 7.34–7.40 (m, 3H, 2-4-F-Ph), 7.72 (s, 3H, H3). ^13^C-NMR (100 MH_Z_, CDCl_3_, δ; ppm): 95.9 (C4), 105.2 (dd, ^2^*J*_C–F_ = 26, ^2^*J*_C–F_ = 24, CAr), 112.3 (dd, ^2^*J*_C–F_ = 23, ^4^*J*_C–F_ = 4, CAr), 124.2 (dd, ^2^*J*_C–F_ = 12, ^4^*J*_C–F_ = 4, CAr) 129.1 (CAr), 129.8 (d, ^3^*J*_C–F_ = 10, CAr), 131.3 (CAr), 140.9 (C5), 142.1 (C3), 156.6 (dd, ^1^*J*_C–F_ = 256, ^3^*J*_C–F_ = 13, CAr), 162.9 (dd, ^1^*J*_C–F_ = 253, ^3^*J*_C–F_ = 11, CAr). Positive ESI-MS *m*/*z* 850.7 [M + H]^+^.

*1,3,5-Tris(4-iodo-1-(2,4-difluorophenyl)-1H-pyrazol-5-yl)benzene* **10c**. Yield 90%, m.p. 188–189 °C. ^1^H-NMR (400 MH_Z_, CDCl_3_, δ; ppm): 6.71–6.76 (m, 3H, 2-4-F-Ph), 6.88-6.93 (m, 3H, 2-4-F-Ph), 7.10 (s, 3H, HBz), 7.30–7.36 (m, 3H, 2-4-F-Ph), 7.76 (s, 3H, H3). ^13^C-NMR (100 MH_Z_, CDCl_3_, δ; ppm): 62.2 (C4), 105.0 (dd, ^2^*J*_C–F_ = 26, ^2^*J*_C–F_ = 24, CAr), 112.1 (dd, ^2^*J*_C–F_ = 23, ^4^*J*_C–F_ = 4, CAr), 124.0 (dd, ^2^*J*_C–F_ = 12, ^4^*J*_C–F_ = 4, CAr) 129.7 (d, ^3^*J*_C–F_ = 10, CAr), 130.0 (CAr), 131.2 (CAr), 144.2 (C5), 146.1 (C3), 156.5 (dd, ^1^*J*_C–F_ = 256, ^3^*J*_C–F_ = 13, CAr), 162.8 (dd, ^1^*J*_C–F_ = 253, ^3^*J*_C–F_ = 11, CAr). Positive ESI-MS *m*/*z* 990.7 [M + H]^+^.

*1,3,5-Tris(4-chloro-1-(2,4-dichlorophenyl)-1H-pyrazol-5-yl)benzene* **11a**. Yield 81%, m.p. 206–207 °C. ^1^H-NMR (400 MH_Z_, CDCl_3_, δ; ppm): 7.15 (s, 3H, HBz), 7.25 (s, 1H, 2-4-Cl-Ph), 7.27 (s, 2H, 2-4-Cl-Ph), 7.31 (d, ^3^*J *= 2, 2H, 2-4-Cl-Ph), 7.33 (d, ^3^*J *= 2, 1H, 2-4-Cl-Ph), 7.41 (d, ^3^*J *= 2, 3H, 2-4-Cl-Ph), 7.70 (s, 3H, H3). ^13^C-NMR (100 MH_Z_, CDCl_3_, δ; ppm): 110.8 (C4), 128.4 (CAr), 128.5 (CAr), 130.5 (CAr), 130.6 (CAr), 130.6 (CAr), 132.9 (CAr), 136.0 (CAr), 136.3 (CAr), 138.9 (C5), 140.0 (C3). Positive ESI-MS *m*/*z* 814.7 [M + H]^+^.

*1,3,5-Tris(4-Bromo-1-(2,4-dichlorophenyl)-1H-pyrazol-5-yl)benzene* **11b**. Yield 82%, m.p. 208–209 °C. ^1^H-NMR (400 MH_Z_, CDCl_3_, δ; ppm): 7.15 (s, 3H, HBz), 7.20 (s, 1H, 2-4-Cl-Ph), 7.23 (s, 2H, 2-4-Cl-Ph), 7.28 (d, ^3^*J *= 2, 2H, 2-4-Cl-Ph), 7.30 (d, ^3^*J *= 2, 1H, 2-4-Cl-Ph), 7.40 (d, ^3^*J *= 2, 3H, 2-4-Cl-Ph), 7.73 (s, 3H, H3). ^13^C-NMR (100 MH_Z_, CDCl_3_, δ; ppm): 95.8 (C4), 128.3 (CAr), 128.9 (CAr), 130.6 (CAr), 130.6 (CAr), 131.3 (CAr), 132.9 (CAr), 136.0 (CAr), 136.3 (CAr), 140.6 (C5), 142.0 (C3). Positive ESI-MS *m*/*z* 948.5 [M + H]^+^.

*1,3,5-Tris(4-iodo-1-(2,4-dichlorophenyl)-1H-pyrazol-5-yl)benzene* **11c**. Yield 85%, m.p. 209–210 °C. ^1^H-NMR (400 MH_Z_, CDCl_3_, δ; ppm): 7.08 (s, 3H, HBz), 7.15 (s, 1H, 2-4-Cl-Ph), 7.17 (s, 2H, 2-4-Cl-Ph), 7.25 (d, ^3^*J *= 2, 2H, 2-4-Cl-Ph), 7.28 (d, ^3^*J *= 2, 1H, 2-4-Cl-Ph), 7.39 (d, ^3^*J *= 2, 3H, 2-4-Cl-Ph), 7.78 (s, 3H, H3). ^13^C-NMR (100 MH_Z_, CDCl_3_, δ; ppm): 62.0 (C4), 128.0 (CAr), 129.9 (CAr), 130.5 (CAr), 132.3 (CAr), 132.9 (CAr), 135.9 (CAr), 136.3 (CAr), 144.0 (C5), 146.2 (C3). Positive ESI-MS *m*/*z* 1088.6 [M + H]^+^.

### 3.3. X-ray Crystallography

Single crystals suitable for X-ray diffraction were obtained in CHCl_3_/EtOH (1:1), EtOH, and CHCl_3_ for compounds **5**, **7**, and **9b**, respectively. Diffraction measurements of compounds **7** and **9b** were performed using graphite monochromatized MoKα radiation with λ = 0.71073 Å in a Bruker X8 APEX II diffractometer with charge-coupled device (CCD) detector. Diffraction measurements of compound **5** were carried out using a Bruker D8 Venture with a Photon 100 CMOS detector with graphite-monochromated Mo-Kα radiation (λ = 0.71073 Å). Anisotropic displacement parameters for non-hydrogen atoms were applied. All hydrogen atoms of the aromatic rings and alkyl groups were positioned geometrically (aromatic C-H = 0.93 Å for Csp^2^ atoms; methyl C-H = 0.96 Å for Csp^3^ atoms) and treated as riding on their respective C atoms with *Uiso*(H) values set at 1.2 *UeqCsp^2^* and 1.5 *UeqCsp^3^*, respectively. Integration, scaling correction, and data reduction were performed using BRUKER APEX-II. Absorption corrections were performed using Gaussian and multiscan methods. The structures were solved and refined using the WinGX package [43]. The structures were solved using SHELXS software [44] and refined based on the full-matrix least-squares method using SHELXL software 2016/6 [45]. ORTEP projections of the molecular structures were generated using ORTEP-3 software [43].

### 3.4. Quantum Chemical Calculation

Quantum chemical calculations were performed with the Gaussian 09 software package [46]. Wavefunctions were generated at the ωB97X-D/cc-pVDZ level of theory by single point calculations with geometries obtained from X-ray data. The wavefunctions were subsequently analyzed from the perspective of the quantum theory of atoms in molecules by AIMALL software [47]. Molecular electrostatic potentials were generated with a isosurface value of 0.001 in GaussView software [48].

## 4. Conclusions

Understanding the supramolecular environment of molecular structures is vital to designing new compounds with desired properties. A series of novel 1,3,5-*tris*(1-phenyl-1*H*-pyrazol-5-yl)benzenes with different substituents was presented. A combination of molecular and supramolecular approaches was shown to evaluate the possibility of 1D stacked formation, which may lead to different applications. The single crystal diffraction technique, DFT, and QTAIM analysis were reliable tools to detect differences in molecular and supramolecular conformation. Additionally, liquid-state ^1^H-NMR experiments confirmed that the same behavior observed in the solid-state occurs in solution without the formation of planar stacking interactions.

## Figures and Tables

**Table 1:** QTAIM data and stabilization energies from intramolecular interactions presented in compounds **5**, **7**, and **9a**.

Compound	Molecular Structure	Intramolecular Interaction	ρ (a.u.)	E_INT_^a^ (kcal mol^−1^)
**5**	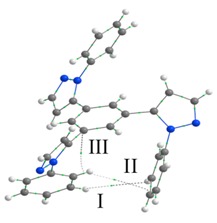	I-CH_B_···π_ph_	0.0017	−0.22
		II-CH_B_···π_ph_	0.0023	−0.33
		III-CH_B_···π_bz_	0.0089	−1.49
**7**	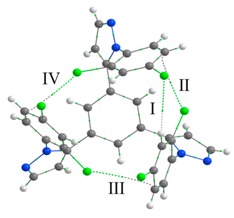	I-CH_A_···Cl_C_	0.0029	−0.44
		II-Cl_B_···π_phA_	0.0061	−0.89
		III-Cl_A_···π_phC_	0.0067	−0.97
		IV-Cl_C_···π_phB_	0.0071	−1.08
**9b**	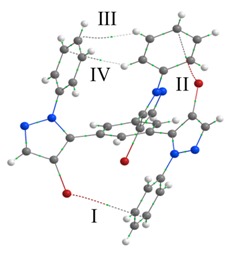	I-Br_C_···π_PhA_	0.0032	−0.42
		II-Br_A_···π_PhB_	0.0042	−0.61
		III-CH_B_···π_PhC_	0.0036	−0.49
		IV-CH_B_···π_PhC_	0.0026	−0.35

^a^ E_INT_ = V/2 [34]. CCPs and RCPs were omitted for clarity purposes.

## Supplementary Materials

Supplementary materials are available online. CCDC numbers 1501480 (**5**), 1484377 (**7**), and 1484578 (**9b**) contain the supplementary crystallographic data for the presented compounds. These data can be obtained free of charge from the Cambridge Crystallographic Data Centre via http://www.ccdc.cam.ac.uk/data_request/cif. Electronic Supplementary Materials (ESM) available: Tables S1–S4, Figures S1–S38.
