# Supplementary material for: Synthesis, Crystal Structure, and Supramolecular Understanding of 1,3,5-Tris(1-phenyl-1H-pyrazol-5-yl)benzenes
Source: Molecules. 2017 Dec 22;23(1):22. doi: 10.3390/molecules23010022 (PMC5943957; doi:10.3390/molecules23010022)
Supplement: Supplementary file 1 [file molecules-23-00022-s001.pdf]

*Supplementary information*

# Synthesis, Crystal Structure and Supramolecular Understanding of 1,3,5-*Tris*(1-phenyl-1*H*-pyrazol-5-yl)benzenes


**Marcos A. P. Martins [1]*, Alexandre R. Meyer [1], Paulo R. S. Salbego [1], Daniel M. dos Santos [1], Guilherme A. de Moraes [2], Helio G. Bonacorso [1], Nilo Zanatta [1], Manfredo Hörner [2]**

[1] Núcleo de Química de Heterociclos (NUQUIMHE), Department of Chemistry, Federal University of Santa Maria (UFSM), 97105-900, Santa Maria, RS, Brazil

[2] Núcleo de Investigação de Triazenos e Complexos (NITRICO), Department of Chemistry, Federal University of Santa Maria (UFSM), 97105-900, Santa Maria, RS, Brazil

* Correspondence: marcos.nuquimhe@gmail.com Tel.: +55 (55) 3220 8756


---

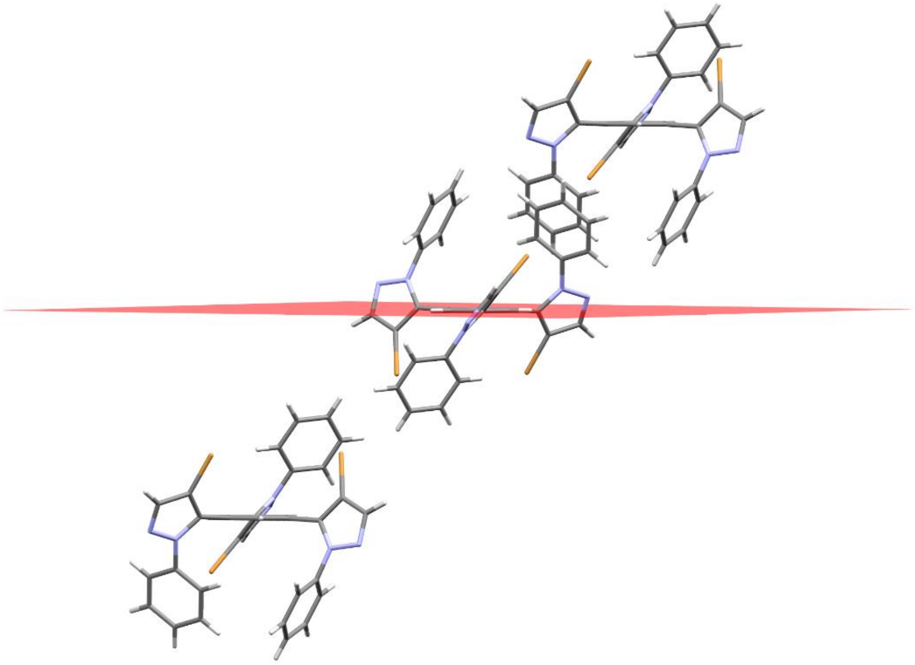

**Figure S1**. 1D arrangement for compound **9b**.

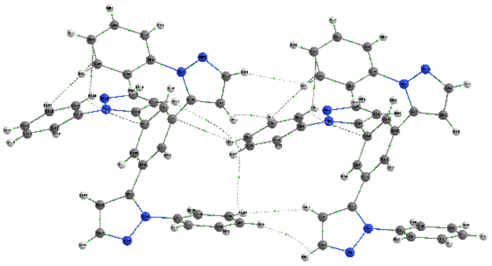

(a)

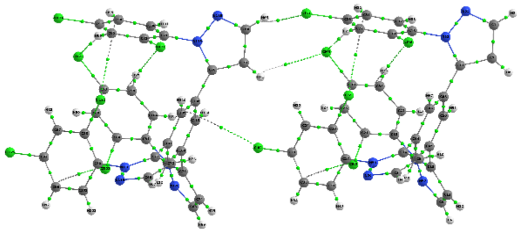

(b)

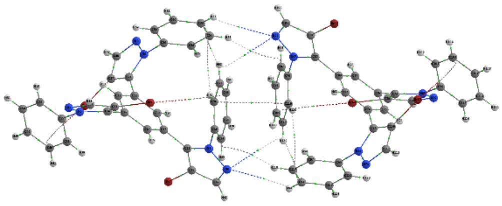

(c)

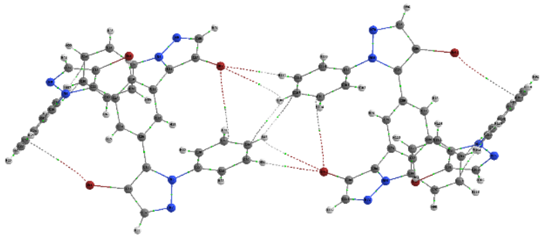

(d)

**Figure S2.** BCPs for the dimers involved in the 1D arrangement for compounds **5** (a), **7** (b), and **9b** (c, d).

**Table S1.** Data collection and structure refinement for structures **5**, **7** and **9b**.

| Compound | (**5**) | (**7**) | (**9b**) |
|---|---|---|---|
| Crystal data | | | |
| Chemical formula | $C_{33} H_{24} N_6$ | $C_{33} H_{18} Cl_6 N_6$ | $C_{33} H_{21} Br_3 N_6$ |
| CCDC number | 1501480 | 1484377 | 1484578 |
| $M_r$ | 504.58 | 711.23 | 741.26 |
| Crystal system, space group | Monoclinic, $P\ 2_1/n$ | Monoclinic, $P\ 2_1/n$ | Triclinic, $P\bar{1}$ |
| Temperature (K) | 293 | 293 | 293 |
| *a, b, c* (Å) | 8.811(7), 31.67(2), 9.691(9) | 10.3914(2), 31.6622(2), 10.5723(2) | 8.7732(3), 10.8040(3), 16.7315(5) |
| α, β, γ (°) | 90, 97.85, 90 | 90, 110.8580(10), 90 | 76.0570(10), 87.256(2), 79.5720(10) |
| V (Å$^3$) | 2679(4) | 3250.48(12) | 1513.74(8) |
| Z | 4 | 4 | 2 |
| F(000) | 1056 | 1440 | 732 |
| $D_x$ (Mg m$^{-3}$) | 1.251 | 1.453 | 1.626 |
| Radiation type | Mo *Kα* | Mo *Kα* | Mo *Kα* |
| μ (mm$^{-1}$) | 0.076 | 0.56 | 4.03 |
| Crystal size (mm) | 0.434 x 0.233 x 0.084 | 0.520 x 0.490 x 0.220 | 0.506 x 0.210 x 0.164 |
| Data collection | | | |
| Diffractometer | X8 APEX II | X8 APEX II | X8 APEX II |
| Absorption correction (Coppens *et al.*, 1965) | Gaussian | Gaussian | Gaussian |
| $T_{min}$, $T_{max}$ | 0.980, 0.994 | 0.873, 0.908 | 0.435, 0.639 |
| Reflections collected, unique | 44903, 5466 | 50606, 7215 | 39956, 6708 |
| $R_{int}$ | 0.123 | 0.035 | 0.052 |
| $\theta_{max}$ (°) | 26.52 | 27.16 | 27.18 |
| Refinement | | | |
| $R[F^2 > 2\sigma(F^2)], wR(F^2), S$ | 0.061, 0.110, 1.00 | 0.082, 0.249, 1.06 | 0.039, 0.087, 1.02 |
| N°. of reflections | 5466 | 7215 | 6708 |
| N°. of parameters | 353 | 406 | 379 |
| $\Delta\varrho_{max}$, $\Delta\varrho_{min}$ (e Å$^{-3}$) | 0.173, −0.190 | 2.563[a], −0.408 | 0.440,-0.749 |

[a] Highest peak: 2,536 (e Å$^{-3}$) at 0.6044, -0.0557, 0.8186 [0.761 Å from H16C].

**Table S2.** QTAIM data of intramolecular interactions of compounds **5**, **7**, and **9b**.

| Comp. | Interaction | ρ (a.u.) | $\nabla^2\rho$ (a.u.) | G (a.u.) | V (a.u) | BPL (Å) | ε |
|---|---|---|---|---|---|---|---|
| **5** | $CH_B\cdots\pi_{ph}$ | +0.001709 | +0.005703 | +0.001064 | -0.000703 | +7.029502 | +1.348943 |
| | $CH_B\cdots\pi_{ph}$ | +0.002334 | +0.007711 | +0.001482 | -0.001036 | +6.764544 | +0.735388 |
| | $CH_B\cdots\pi_{bz}$ | +0.008895 | +0.036788 | +0.006966 | -0.004735 | +5.741479 | +0.940599 |
| **7** | $CH_A\cdots Cl_C$ | +0.002861 | +0.009384 | +0.001869 | -0.001392 | +6.295619 | +0.069233 |
| | $Cl_B\cdots\pi_{phA}$ | +0.006076 | +0.017295 | +0.003573 | -0.002822 | +6.775708 | +1.182899 |
| | $Cl_A\cdots\pi_{phC}$ | +0.006672 | +0.019205 | +0.003951 | -0.003100 | +6.850917 | +1.548697 |
| | $Cl_C\cdots\pi_{phB}$ | +0.007134 | +0.021533 | +0.004408 | -0.003432 | +6.503711 | +1.295082 |
| **9b** | $CH_B\cdots\pi_{PhC}$ | +0.002607 | +0.008188 | +0.001588 | -0.001129 | +6.239854 | +0.810114 |
| | $Br_C\cdots\pi_{PhA}$ | +0.003151 | +0.007538 | +0.001608 | -0.001331 | +7.876611 | +1.152721 |
| | $CH_B\cdots\pi_{PhC}$ | +0.003635 | +0.012417 | +0.002331 | -0.001559 | +5.851308 | +0.247889 |
| | $Br_A\cdots\pi_{PhB}$ | +0.004203 | +0.010958 | +0.002338 | -0.001936 | +7.214326 | +2.528119 |

**Table S3.** Geometric data of intramolecular interactions of compounds **5**, **7**, and **9b**.

| Comp. | Molecular Structure | Intramolecular Interaction | Atom-atom distance (Å) | Interaction angle (°) | Atom-centroid distance (Å) |
|---|---|---|---|---|---|
| **5** | 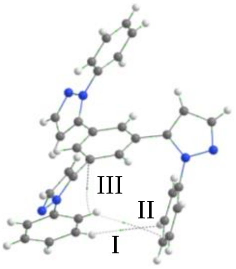  | I $CH_B\cdots\pi_{ph}$ | 3.448 | 130.56 | 4.213 |
| | | II $CH_B\cdots\pi_{ph}$ | 3.362 | 129.51 | 3.411 |
| | | III $CH_B\cdots\pi_{bz}$ | 2.684 | 108.43 | 3.218 |
| **7** | 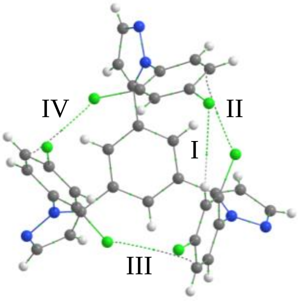  | I $CH_A\cdots Cl_C$ | 3.294 | 156.89 | - |
| | | II $Cl_B\cdots\pi_{phA}$ | 3.445 | 131.58 | 3.609 |
| | | III $Cl_A\cdots\pi_{phC}$ | 3.469 | 141.54 | 3.688 |
| | | IV $Cl_C\cdots\pi_{phB}$ | 3.377 | 142.53 | 3.456 |
| **9b** | 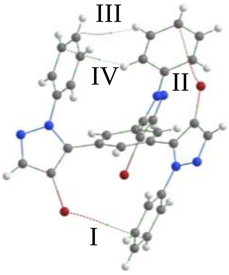  | I $Br_C\cdots\pi_{PhA}$ | 4.006 | 123.71 | 4.326 |
| | | II $Br_A\cdots\pi_{PhB}$ | 3.778 | 151.45 | 3.789 |
| | | III $CH_B\cdots\pi_{PhC}$ | 2.993 | 142.63 | 3.788 |
| | | IV $CH_B\cdots\pi_{PhC}$ | 3.252 | 135.12 | 3.560 |

**Table S4.** QTAIM data of intermolecular interactions for the dimers involved in the 1D arrangement for compounds **5** (a), **7** (b), and **9b** (c, d).

| Comp. | Interaction | ρ (au) | ∇2ρ (a.u.) | ε | K (a.u.) | V (a.u.) | G (a.u.) | BPL (Å) |
|---|---|---|---|---|---|---|---|---|
| **5** | C13$_{C}$-H13$_{C}$···H4$_{C}$-C4$_{C}$ | 0.000625 | +0.002109 | 0.385237 | -0.000166 | -0.000196 | +0.000361 | 7.917284 |
| | C14$_{C}$-H14$_{C}$···H13$_{A}$-C13$_{A}$ | 0.001150 | +0.003850 | 10.87582 | -0.000286 | -0.000391 | +0.000677 | 7.159302 |
| | C4$_{A}$-H4$_{A}$···H13$_{A}$-C13$_{A}$ | 0.001691 | +0.007862 | 0.228573 | -0.000606 | -0.000753 | +0.001359 | 5.405544 |
| | C15$_{B}$-H15$_{B}$···C3$_{B}$-H3$_{B}$ | 0.001810 | +0.008470 | 0.069926 | -0.000649 | -0.000820 | +0.001469 | 5.340153 |
| | C3$_{A}$-H3$_{A}$···H14$_{A}$-C14$_{A}$ | 0.002610 | +0.010479 | 1.451658 | -0.000755 | -0.001110 | +0.001865 | 5.903538 |
| | C14$_{C}$-H14$_{C}$···C51$_{B}$(π) | 0.003203 | +0.009717 | 0.835703 | -0.000487 | -0.001455 | +0.001942 | 6.392775 |
| | C15$_{C}$-H15$_{C}$···H4$_{B}$-C4$_{B}$ | 0.003941 | +0.016360 | 0.367039 | -0.001078 | -0.001934 | +0.003012 | 5.003101 |
| | Total | 0.015030 | | | | | | |
| **7** | C4$_{A}$-H4$_{A}$···Cl2$_{C}$ | 0.002102 | +0.007701 | 0.396783 | -0.000507 | -0.000912 | +0.001418 | 6.755820 |
| | C3$_{A}$-H3$_{A}$···Cl2$_{A}$ | 0.002453 | +0.008534 | 0.872875 | -0.000557 | -0.001020 | +0.001577 | 7.028604 |
| | Cl2$_{B}$···C51$_{A}$(πBz) | 0.005733 | +0.016697 | 2.503532 | -0.000721 | -0.002732 | +0.003453 | 7.029595 |
| | Total | 0.010288 | | | | | | |
| **9b** (dimer 1) | C14$_{C}$-H14$_{C}$···N2$_{B}$ | 0.001974 | +0.008350 | 0.064986 | -0.000589 | -0.000909 | +0.001498 | 6.305415 |
| | C14$_{C}$-H14$_{C}$···N2$_{B}$ | 0.001974 | +0.008349 | 0.065009 | -0.000589 | -0.000909 | +0.001498 | 6.305415 |
| | C15$_{B}$-H15$_{B}$···N2$_{B}$ | 0.003039 | +0.012084 | 0.473979 | -0.000777 | -0.001467 | +0.002244 | 6.126470 |
| | C15$_{B}$-H15$_{B}$···N2$_{B}$ | 0.003039 | +0.012084 | 0.473798 | -0.000777 | -0.001467 | +0.002244 | 6.126470 |
| | C15$_{C}$-H15$_{C}$···C12$_{B}$ | 0.003828 | +0.013741 | 0.602985 | -0.000879 | -0.001678 | +0.002557 | 6.014947 |
| | C16$_{B}$···C16$_{B}$ | 0.003576 | +0.008290 | 3.038742 | -0.000278 | -0.001517 | +0.001795 | 7.049161 |
| | C15$_{C}$-H15$_{C}$···C12$_{B}$ | 0.003827 | +0.013740 | 0.603086 | -0.000879 | -0.001678 | +0.002556 | 6.015115 |
| | Total | 0.021257 | | | | | | |
| **9b** (dimer 2) | C13$_{A}$-H13$_{A}$···Br3$_{C}$ | 0.004336 | +0.015844 | 0.238205 | -0.000914 | -0.002133 | +0.003047 | 6.078877 |
| | C13$_{A}$-H13$_{A}$···Br3$_{C}$ | 0.004336 | +0.015844 | 0.238206 | -0.000914 | -0.002133 | +0.003047 | 6.078877 |
| | C14$_{A}$-H14$_{A}$···Br3$_{C}$ | 0.003426 | +0.011924 | 1.270349 | -0.000714 | -0.001553 | +0.002267 | 6.625252 |
| | C14$_{A}$-H14$_{A}$···Br3$_{C}$ | 0.003426 | +0.011924 | 1.270401 | -0.000714 | -0.001553 | +0.002267 | 6.625252 |
| | Total | 0.015524 | | | | | | |

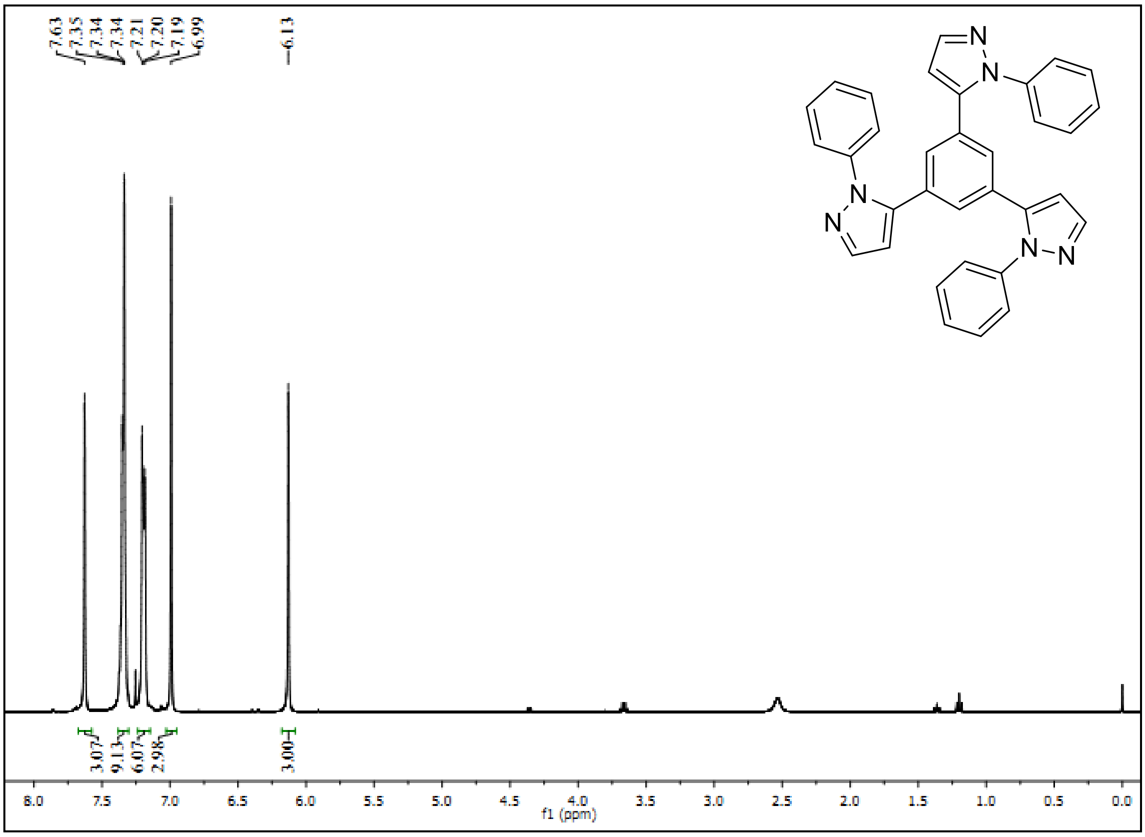

**Figure S3.** $^{1}H$ NMR spectrum of 1,3,5-tris(1-phenyl-1H-pyrazol-5-yl)benzene **5**.

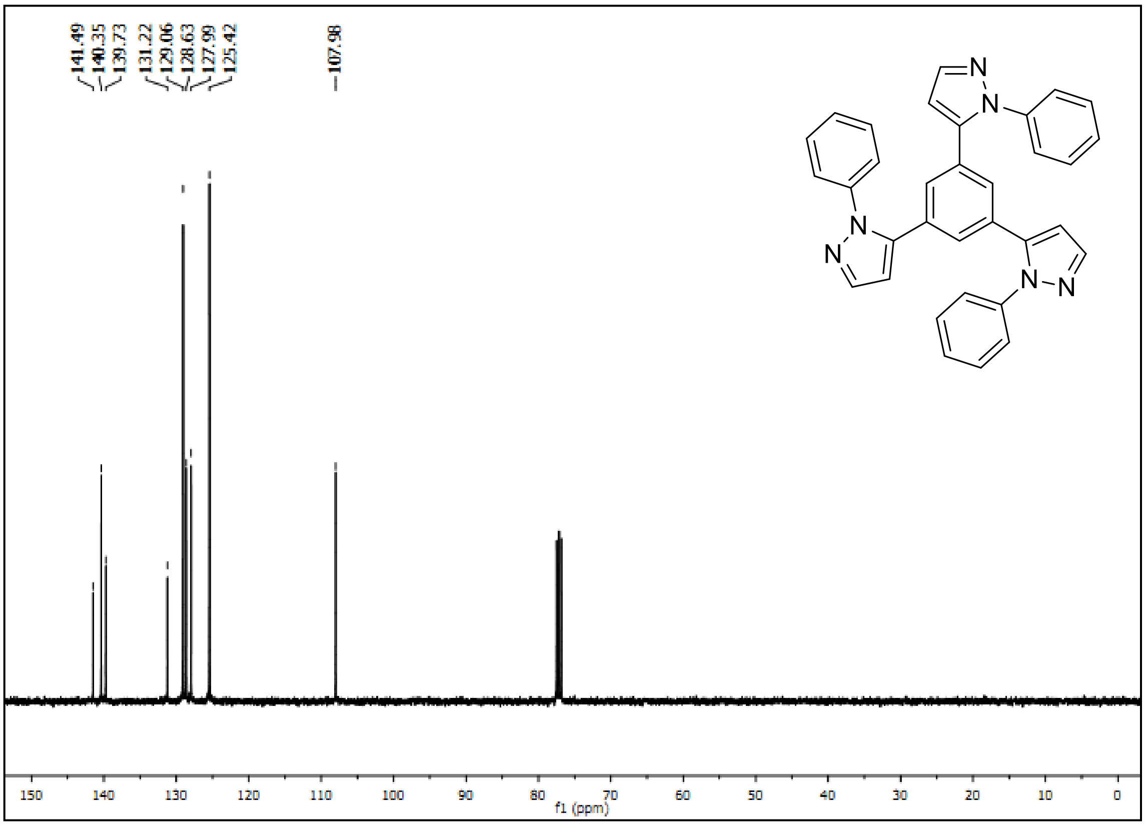

**Figure S4.** $^{13}C$ NMR spectrum of 1,3,5-tris(1-phenyl-1H-pyrazol-5-yl)benzene **5**.

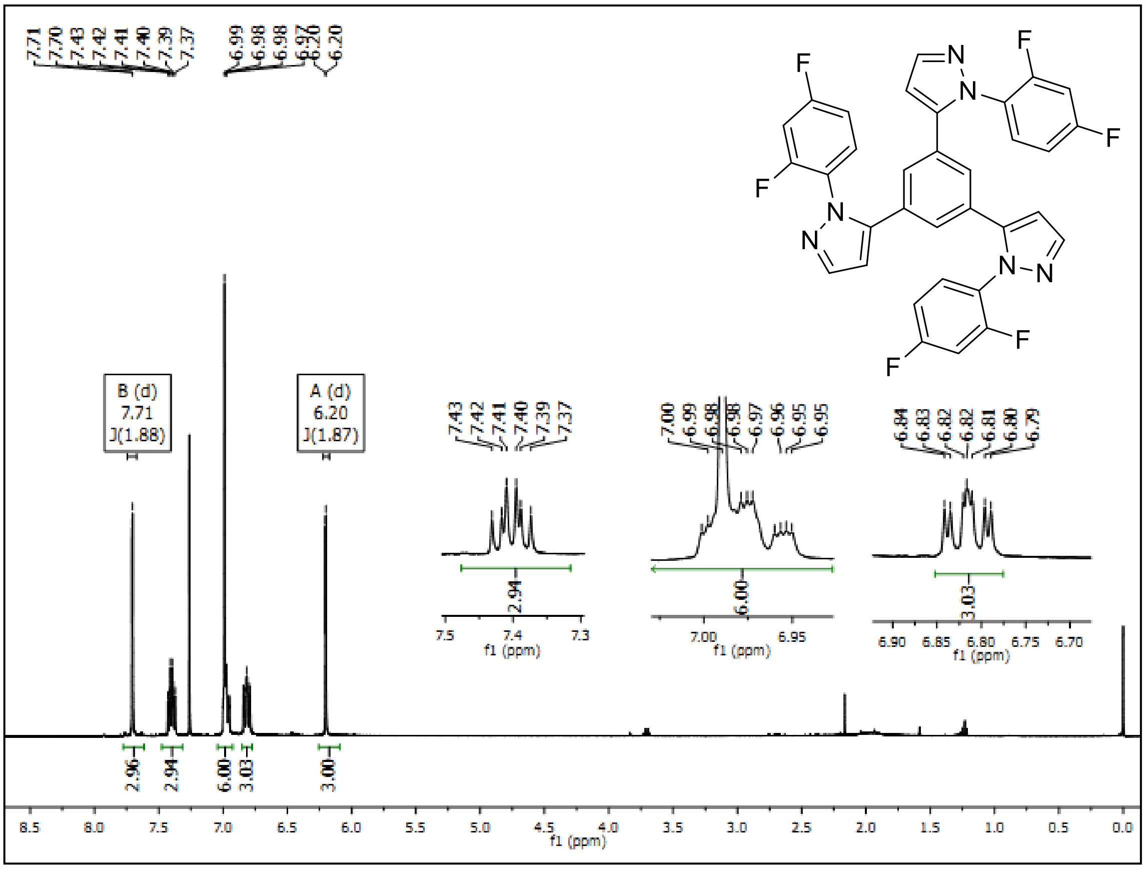

**Figure S5.** $^1$H NMR spectrum of 1,3,5-tris(1-(2,4-difluorophenyl)-1H-pyrazol-5-yl)benzene **6**.

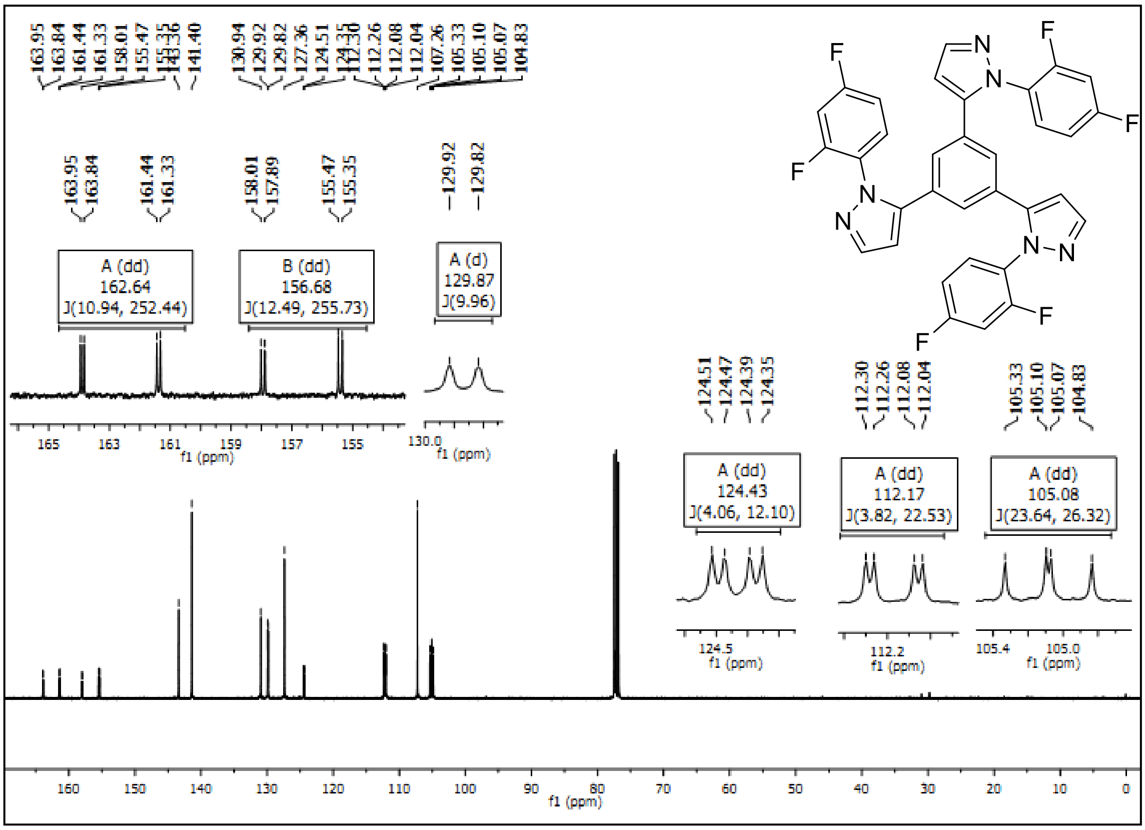

**Figure S6.** $^{13}$C NMR spectrum of 1,3,5-tris(1-(2,4-difluorophenyl)-1H-pyrazol-5-yl)benzene **6**.

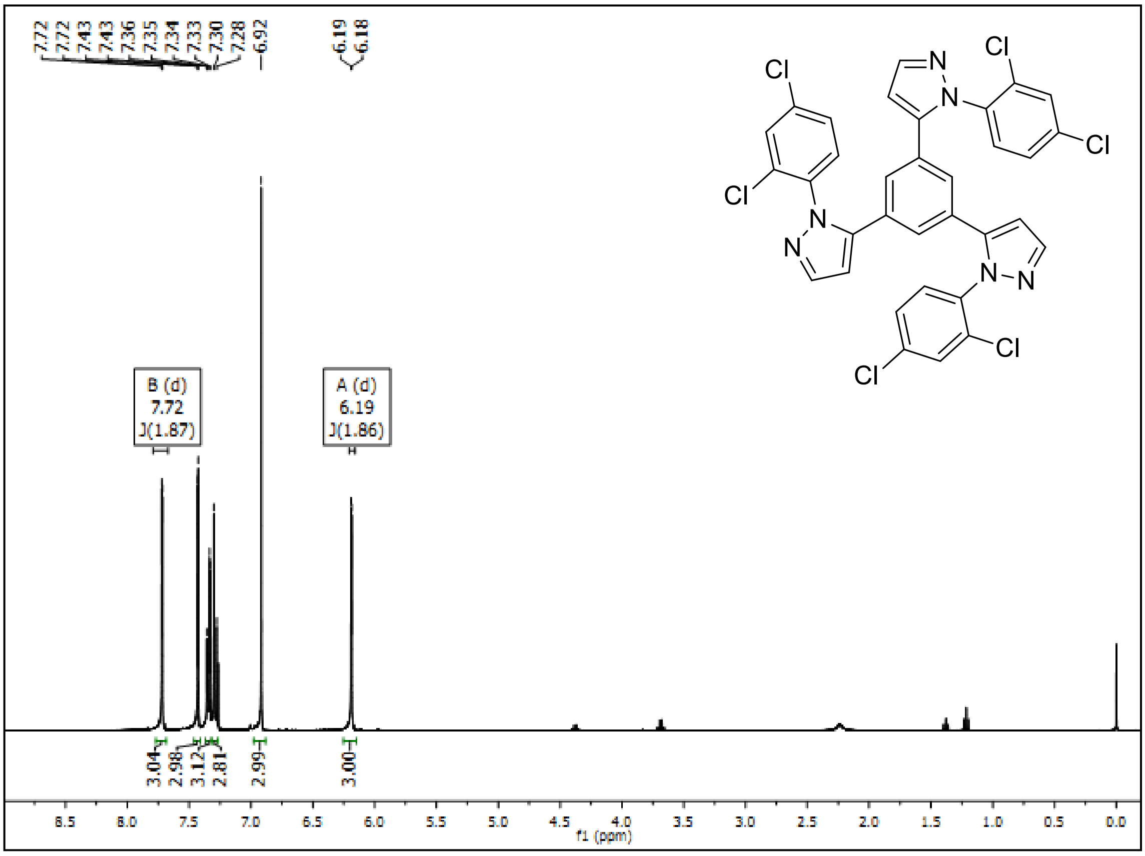


**Figure S7.** $^{1}$H NMR spectrum of 1,3,5-tris(1-(2,4-dichlorophenyl)-1H-pyrazol-5-yl)benzene **7**.

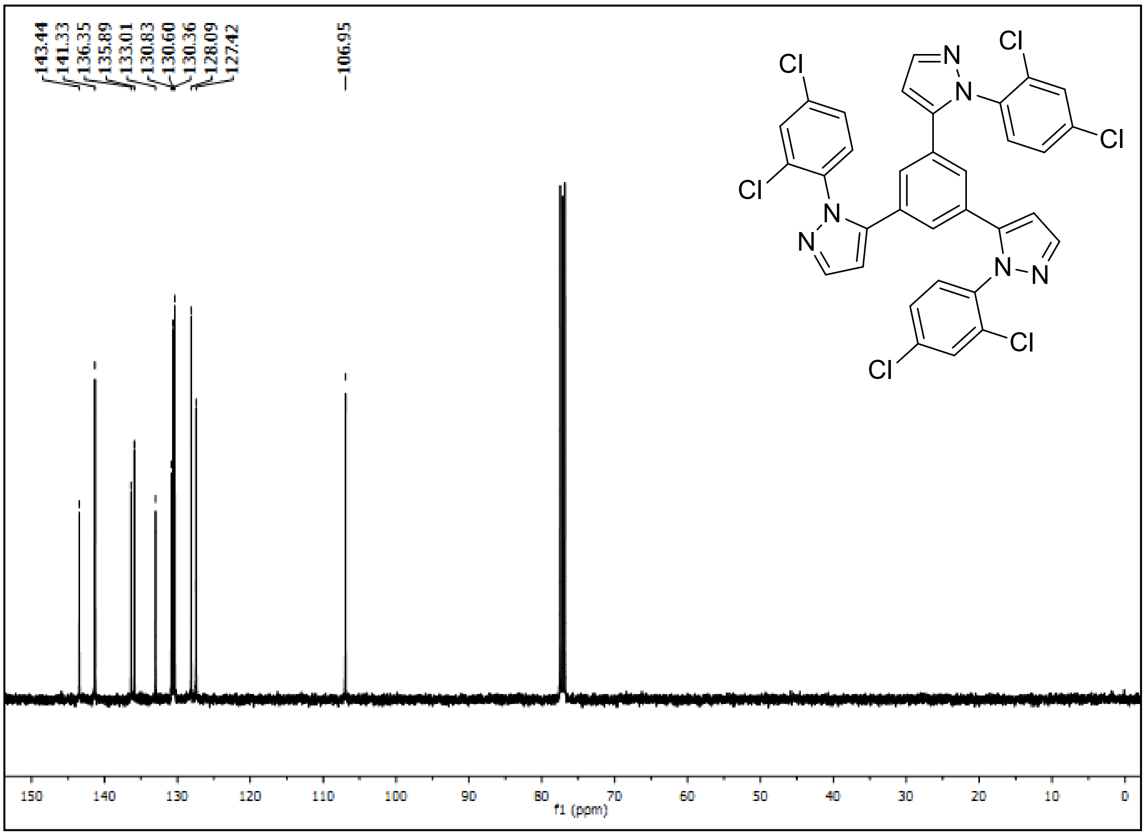


**Figure S8.** $^{13}$C NMR spectrum of 1,3,5-tris(1-(2,4-dichlorophenyl)-1H-pyrazol-5-yl)benzene **7**.

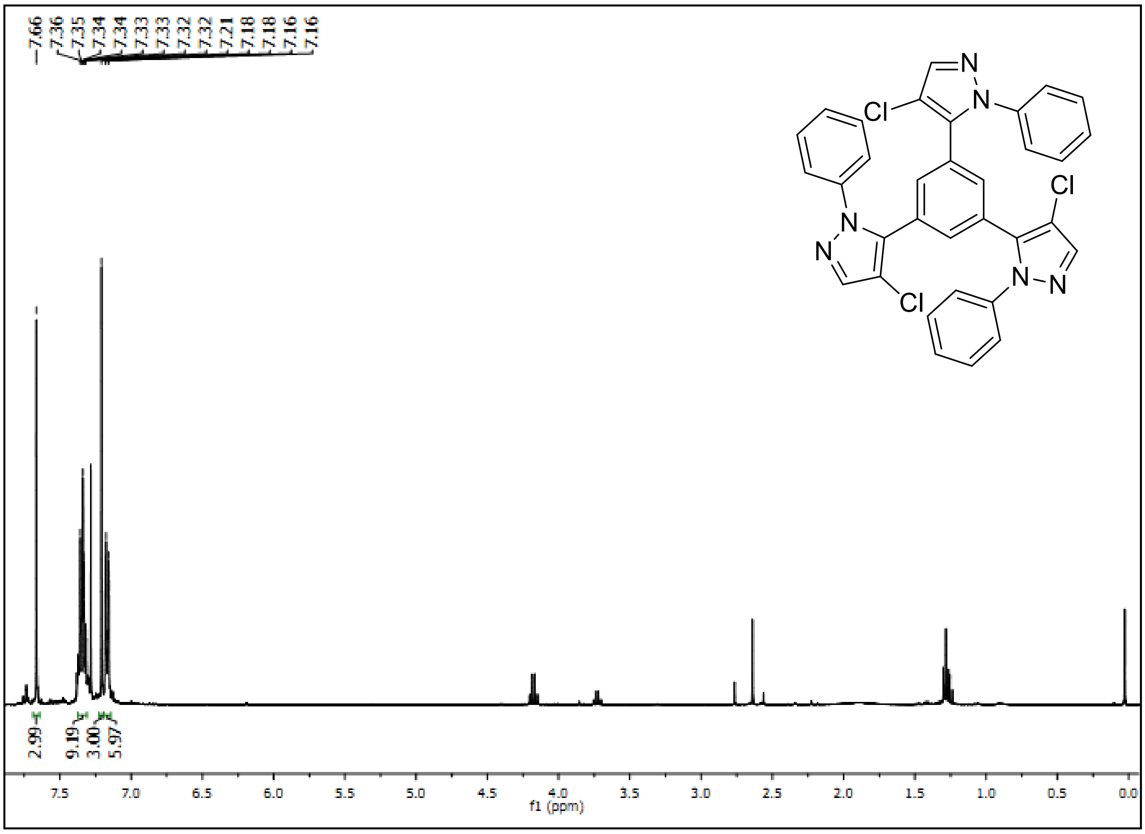

**Figure S9.** $^{1}$H NMR spectrum of 1,3,5-tris(4-chloro-1-phenyl-1H-pyrazol-5-yl)benzene **9a**.

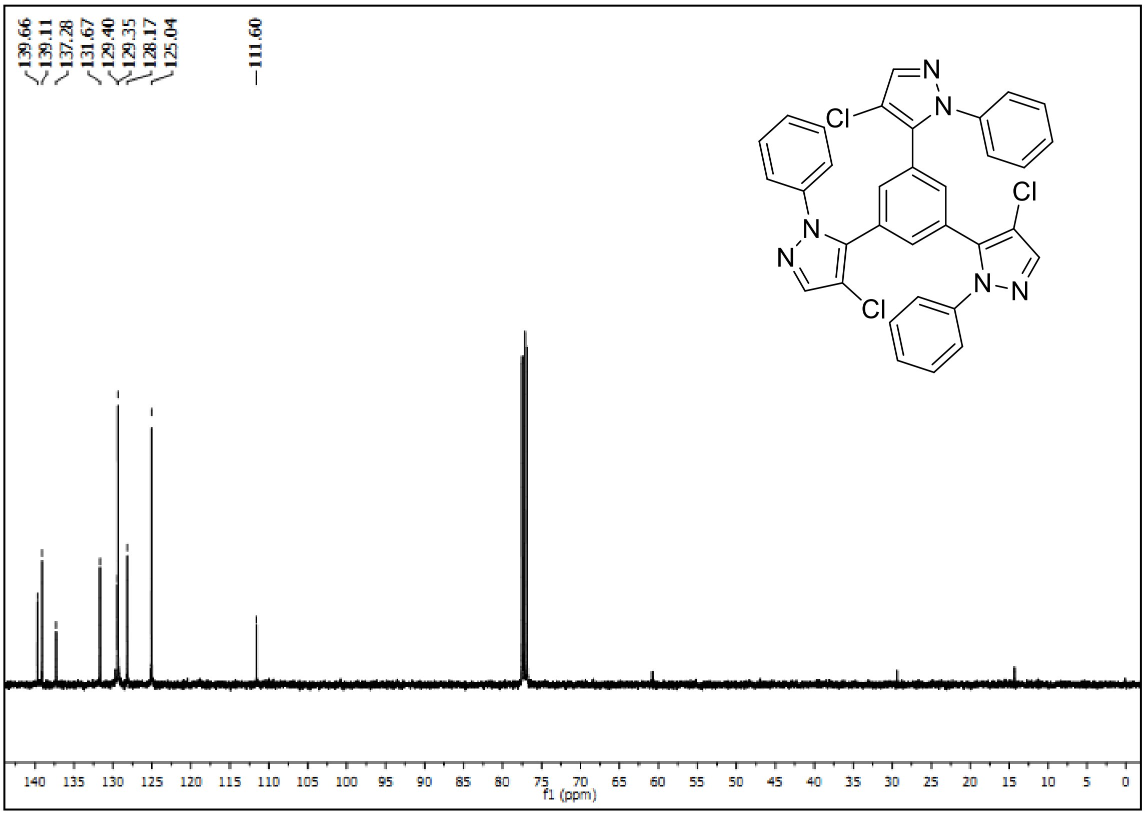

**Figure S10.** $^{13}$C NMR spectrum of 1,3,5-tris(4-chloro-1-phenyl-1H-pyrazol-5-yl)benzene **9a**.

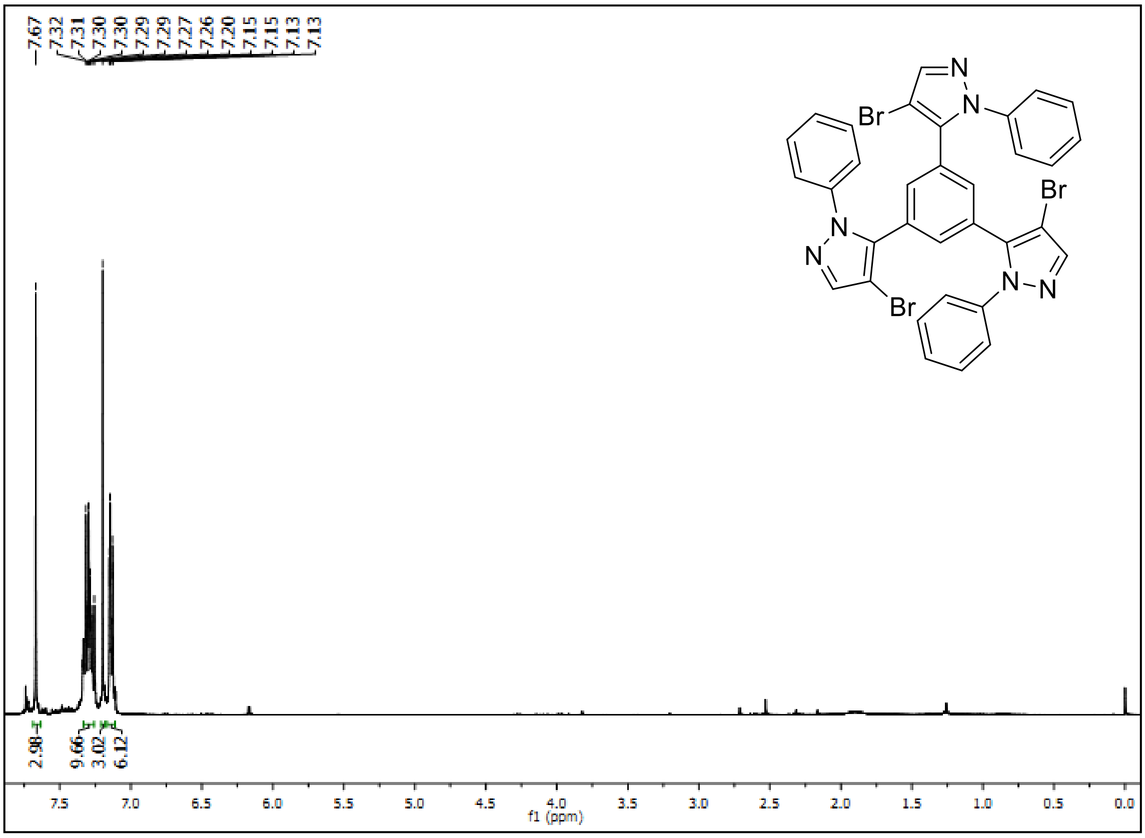

**Figure S11.** $^{1}H$ NMR spectrum of 1,3,5-tris(4-bromo-1-phenyl-1H-pyrazol-5-yl)benzene **9b**.

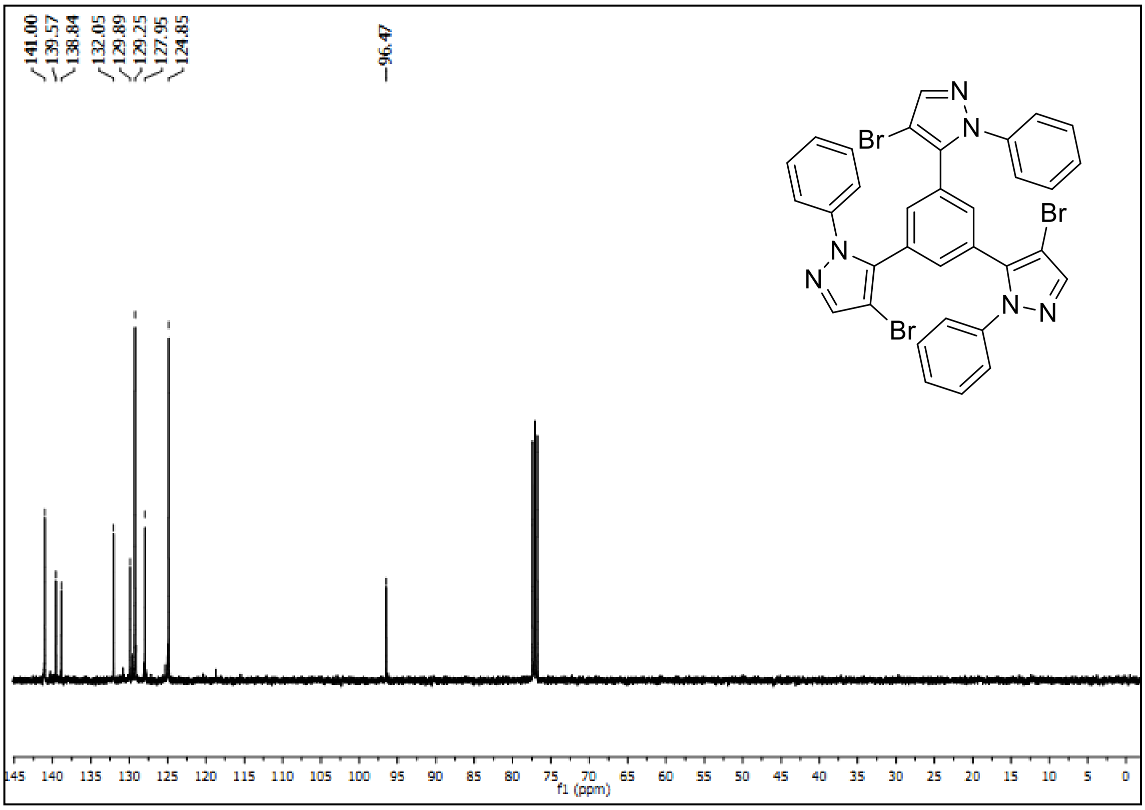

**Figure S12.** $^{13}C$ NMR spectrum of 1,3,5-tris(4-bromo-1-phenyl-1H-pyrazol-5-yl)benzene **9b**.

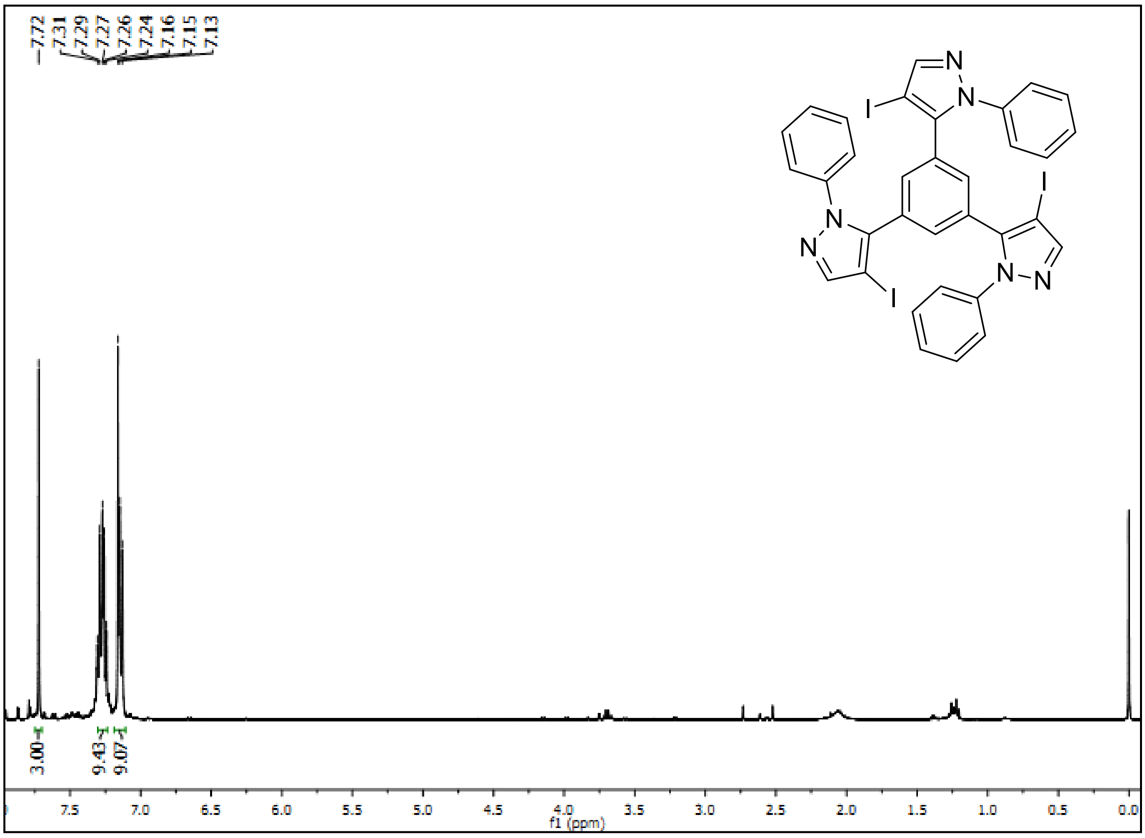

**Figure S13.** $^{1}H$ NMR spectrum of 1,3,5-tris(4-iodo-1-phenyl-1H-pyrazol-5-yl)benzene **9c**.

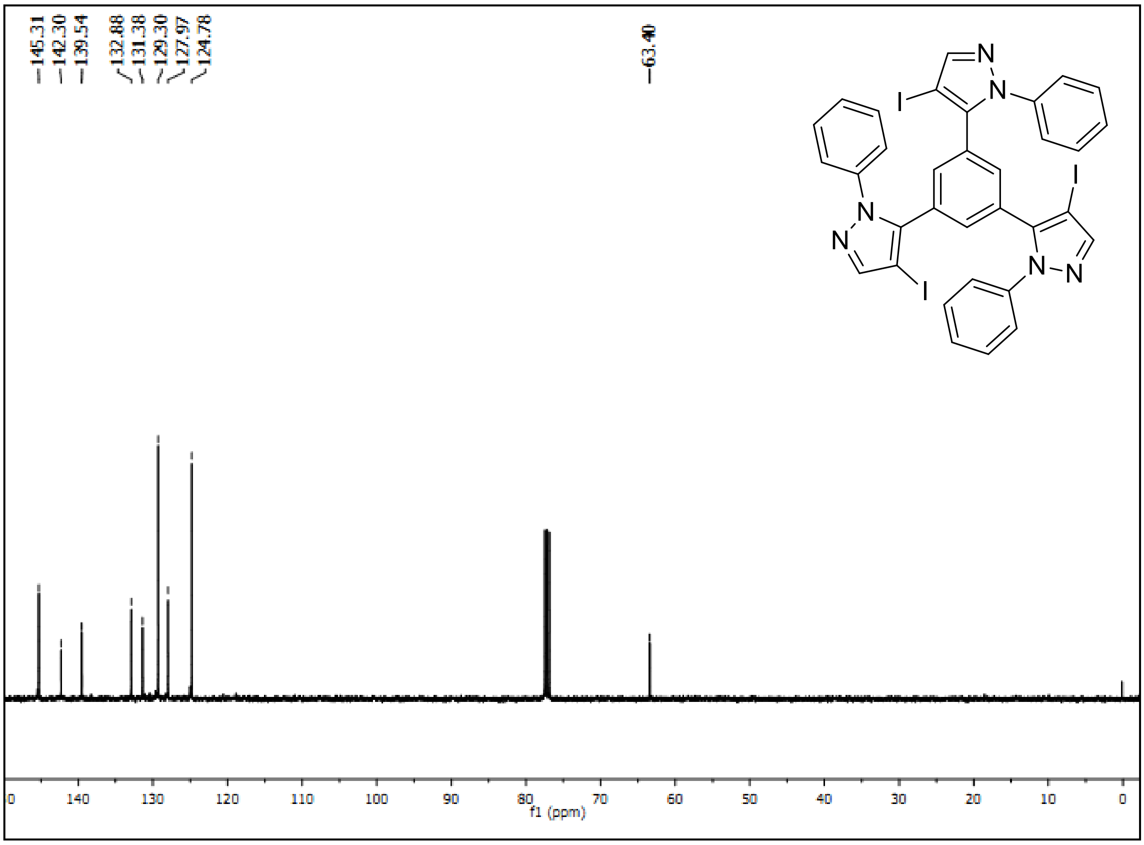

**Figure S14.** $^{13}C$ NMR spectrum of 1,3,5-tris(4-iodo-1-phenyl-1H-pyrazol-5-yl)benzene **9c**.

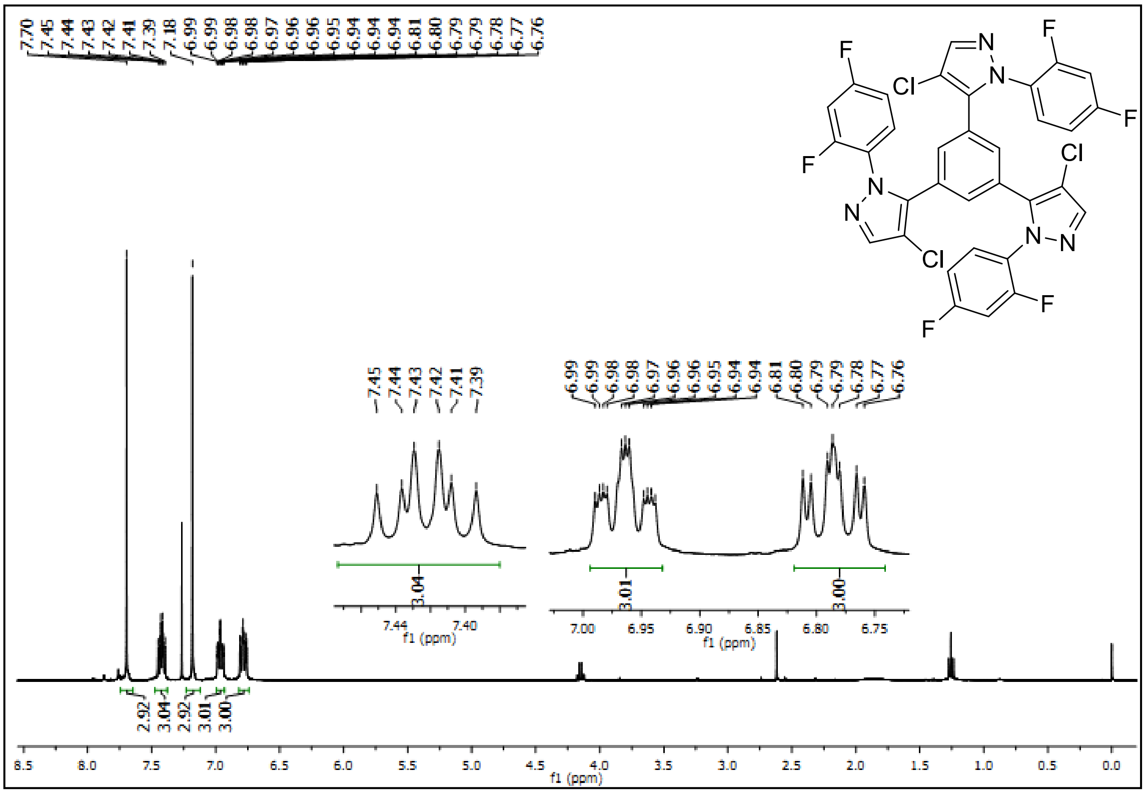

**Figure S15.** $^{1}H$ NMR spectrum of 1,3,5-tris(4-chloro-1-(2,4-difluorophenyl)-1H-pyrazol-5-yl)benzene **10a**.

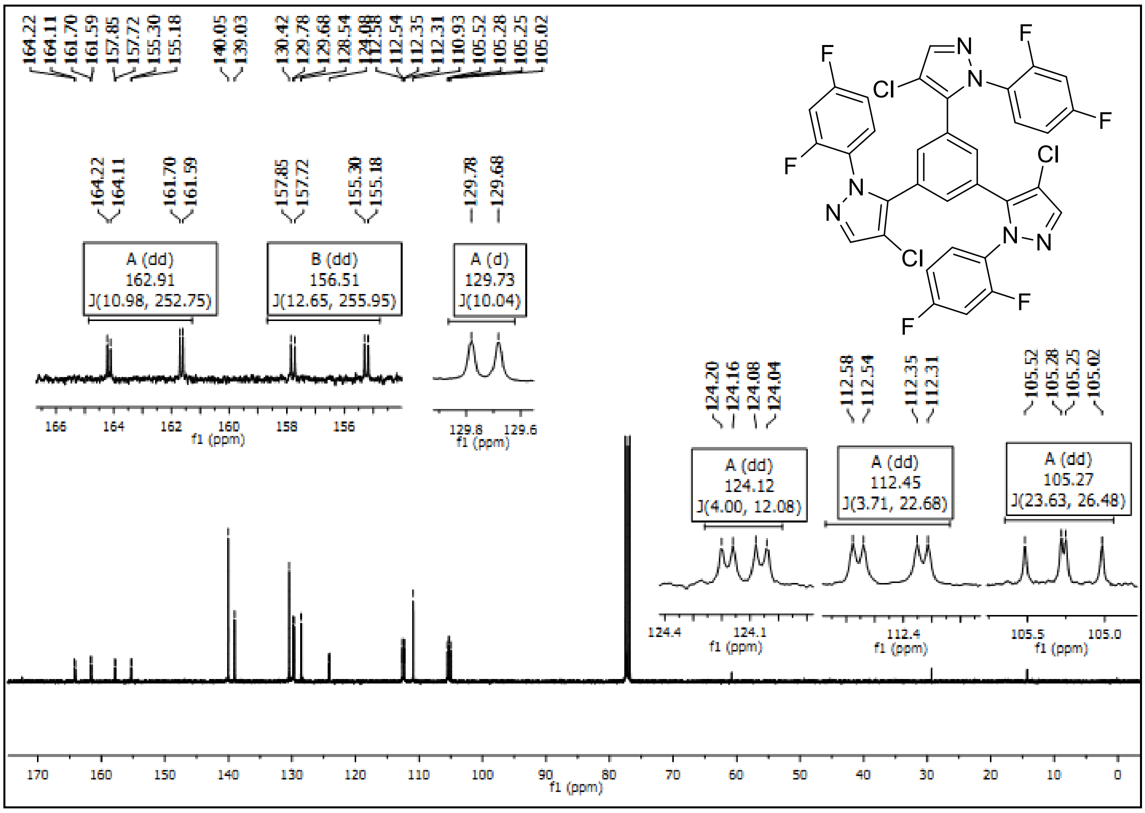


**Figure S16.** $^{13}C$ NMR spectrum of 1,3,5-tris(4-chloro-1-(2,4-difluorophenyl)-1H-pyrazol-5-yl)benzene **10a**.

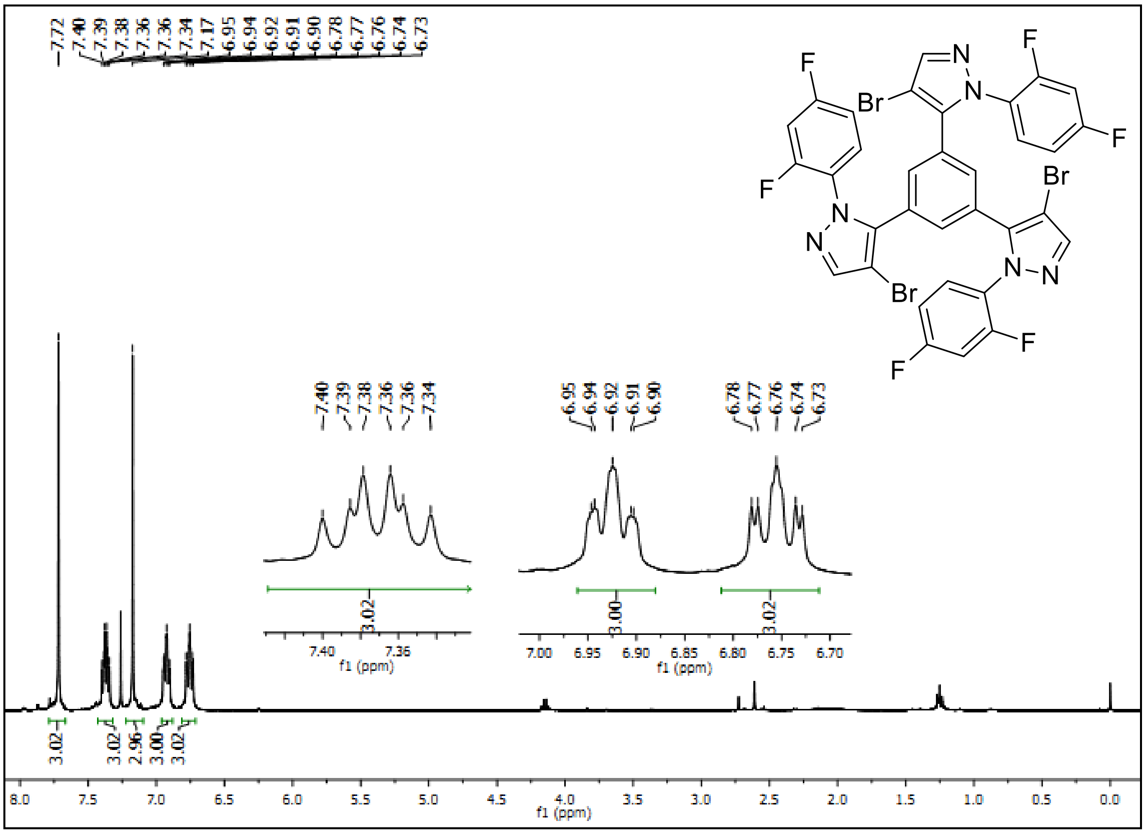

**Figure S17.** $^{1}$H NMR spectrum of 1,3,5-tris(4-bromo-1-(2,4-difluorophenyl)-1H-pyrazol-5-yl)benzene **10b**.

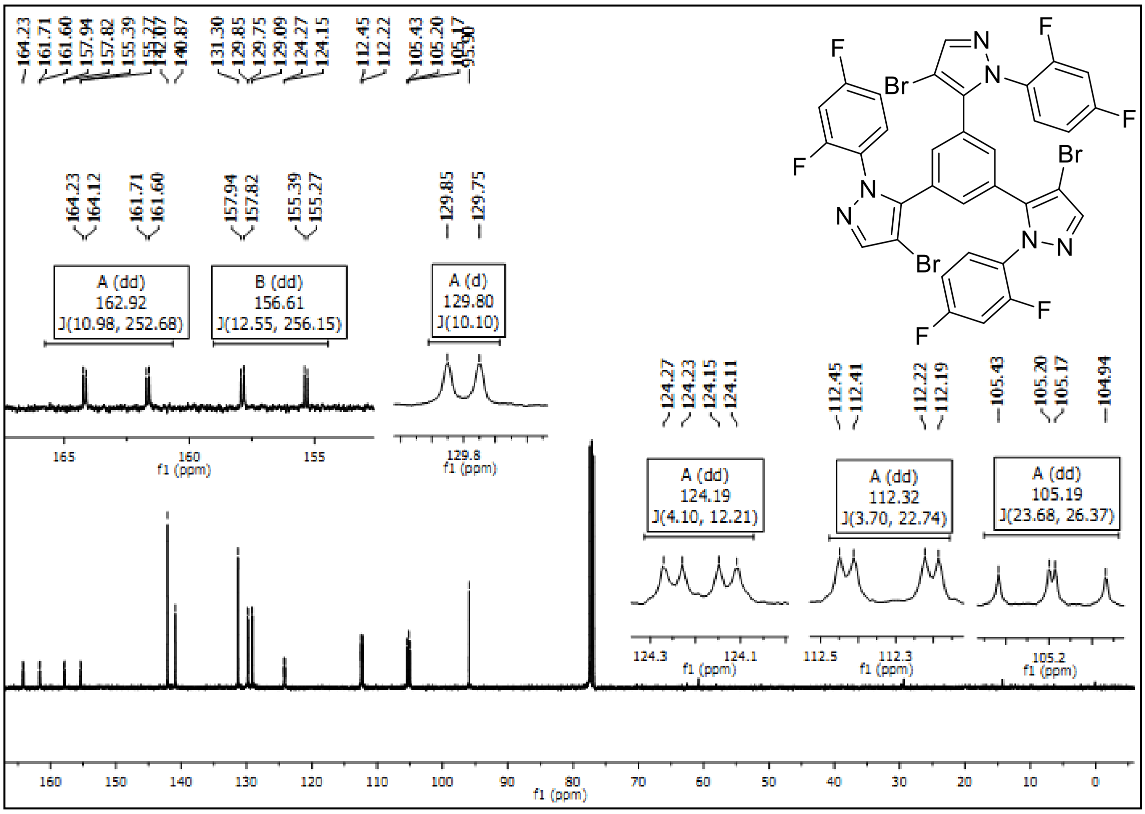

**Figure S18.** $^{13}$C NMR spectrum of 1,3,5-tris(4-bromo-1-(2,4-difluorophenyl)-1H-pyrazol-5-yl)benzene **10b**.

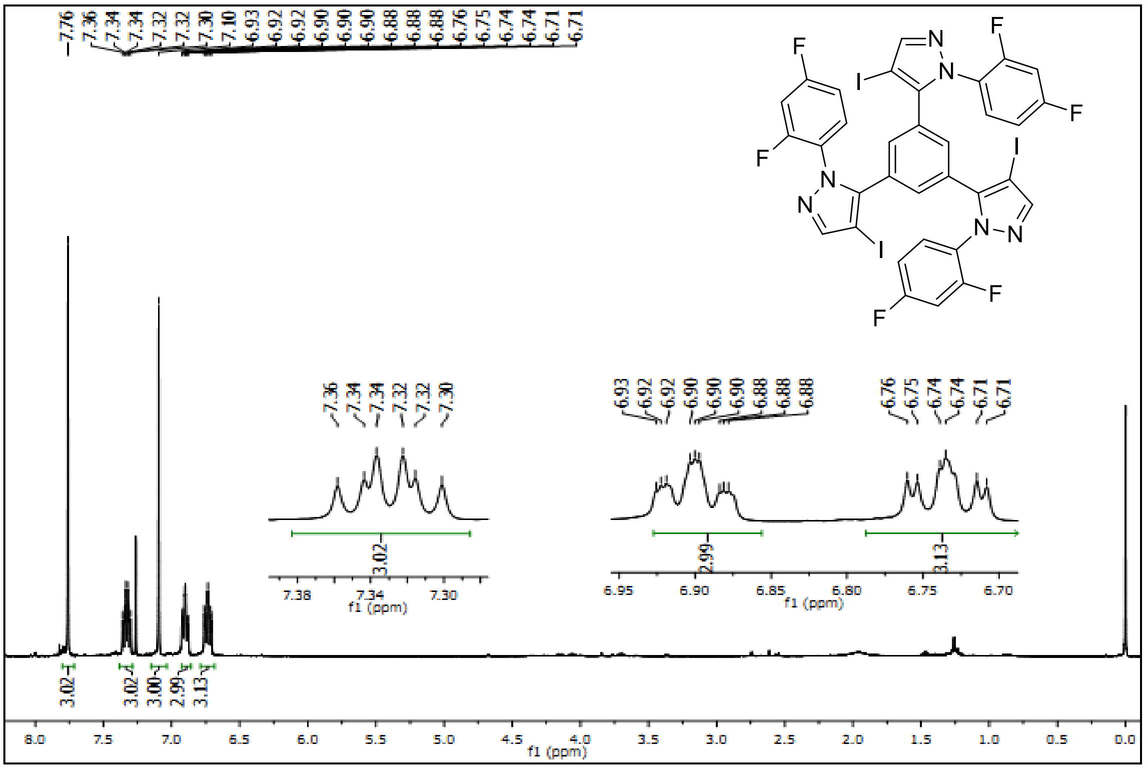

**Figure S19.** $^1$H NMR spectrum of 1,3,5-tris(4-iodo-1-(2,4-difluorophenyl)-1H-pyrazol-5-yl)benzene **10c**.

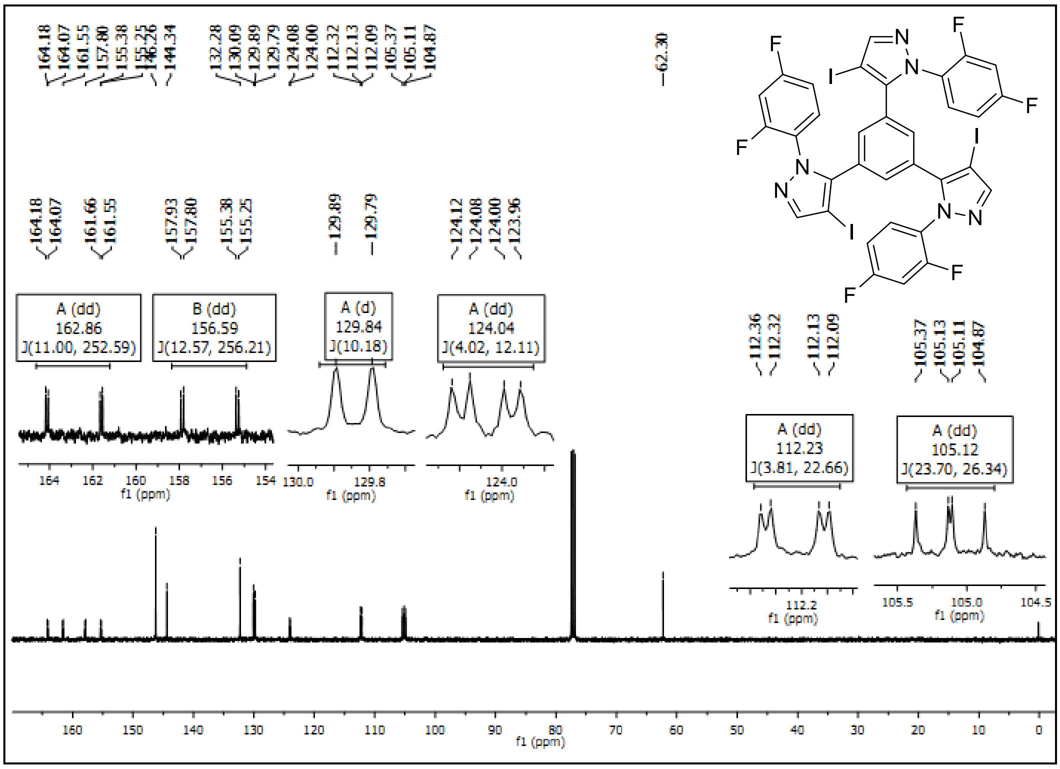

**Figure S20.** $^{13}$C NMR spectrum of 1,3,5-tris(4-iodo-1-(2,4-difluorophenyl)-1H-pyrazol-5-yl)benzene **10c**.

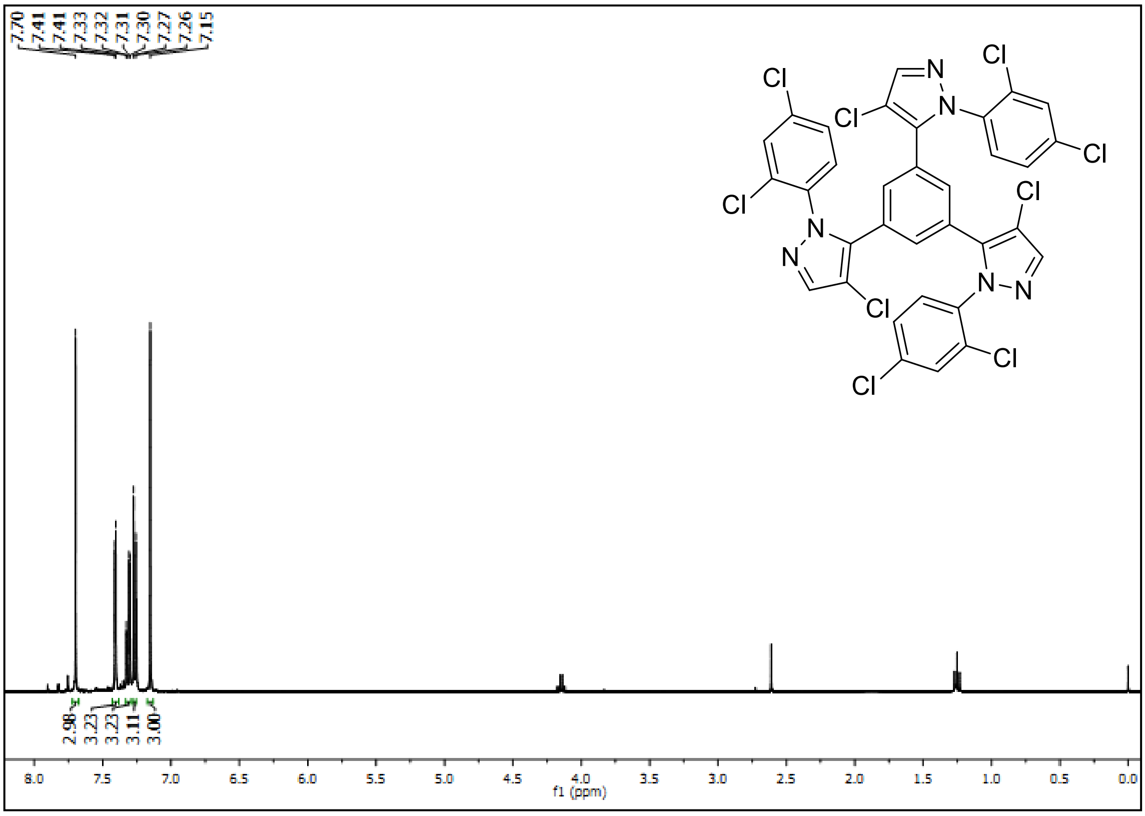

**Figure S21.** $^{1}H$ NMR spectrum of 1,3,5-tris(4-chloro-1-(2,4-dichlorophenyl)-1H-pyrazol-5-yl)benzene **11a**.

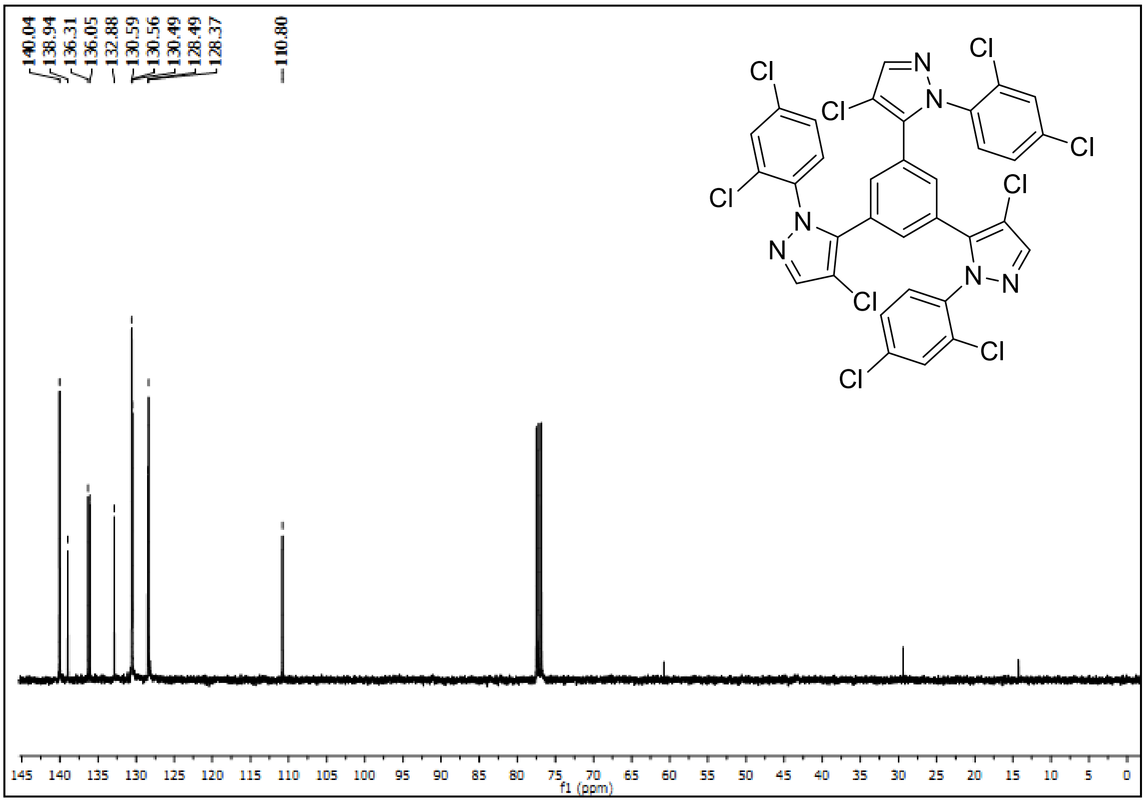

**Figure S22.** $^{13}C$ NMR spectrum of 1,3,5-tris(4-chloro-1-(2,4-dichlorophenyl)-1H-pyrazol-5-yl)benzene **11a**.

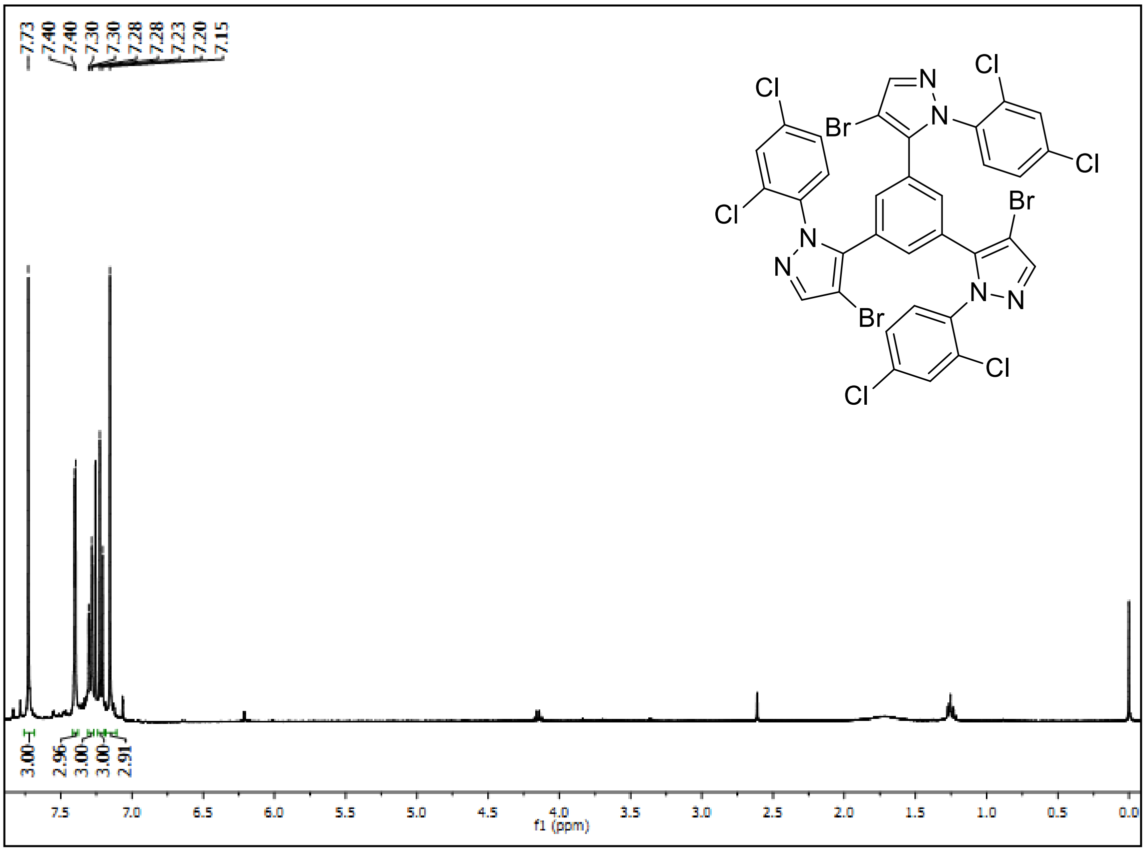


**Figure S23.** $^{1}$H NMR spectrum of 1,3,5-tris(4-Bromo-1-(2,4-dichlorophenyl)-1H-pyrazol-5-yl)benzene **11b**.

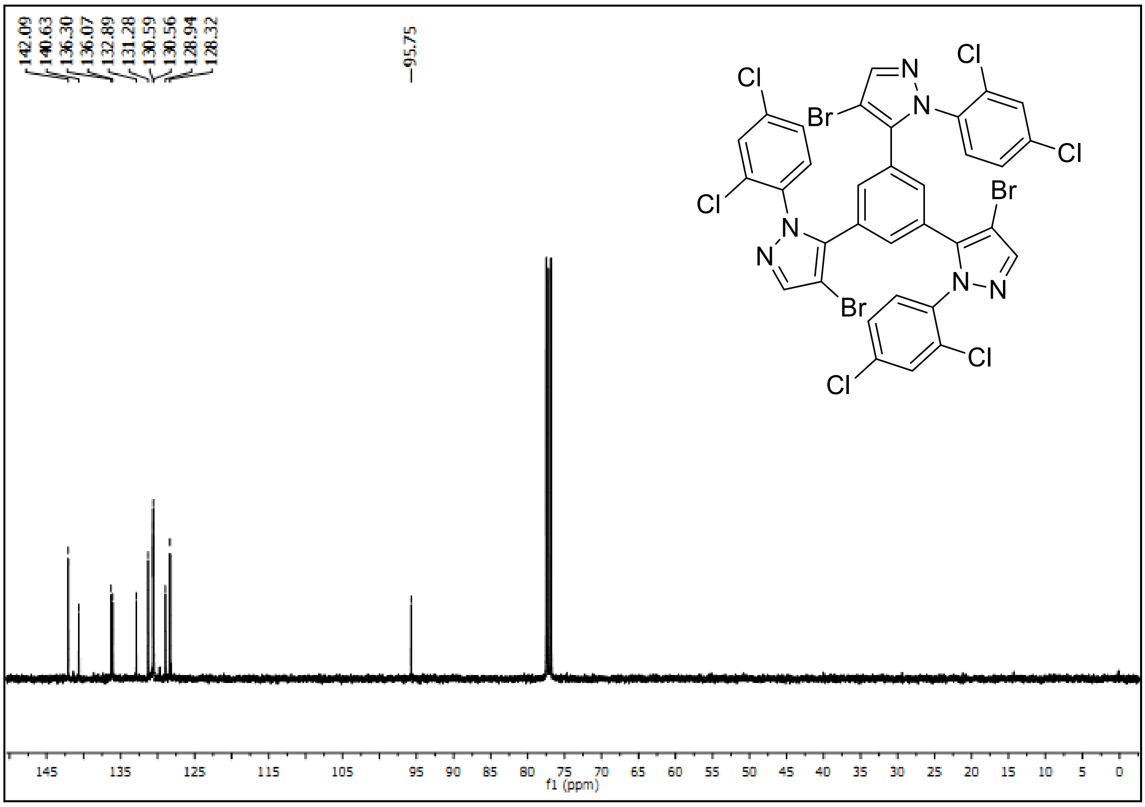


**Figure S24.** $^{13}$C NMR spectrum of 1,3,5-tris(4-Bromo-1-(2,4-dichlorophenyl)-1H-pyrazol-5-yl)benzene **11b**.

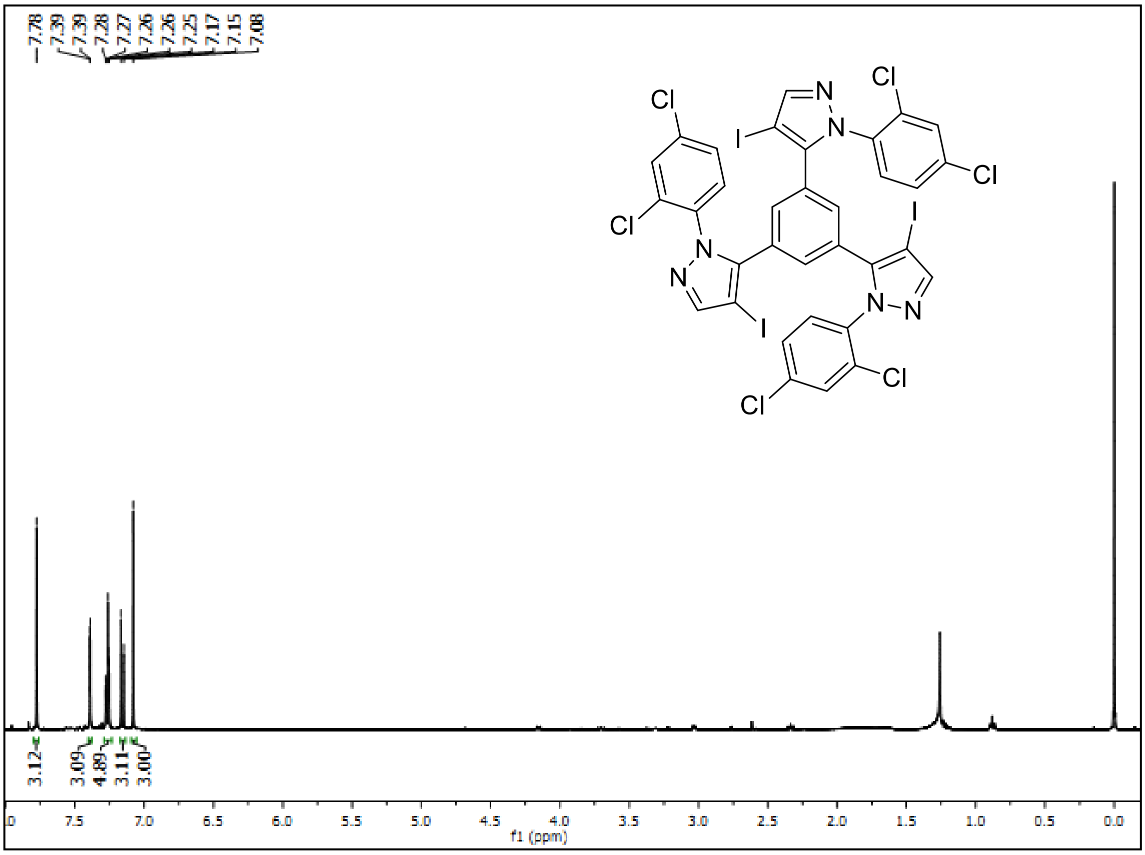


**Figure S25.** ¹H NMR spectrum of 1,3,5-tris(4-iodo-1-(2,4-dichlorophenyl)-1H-pyrazol-5-yl)benzene **11c**.

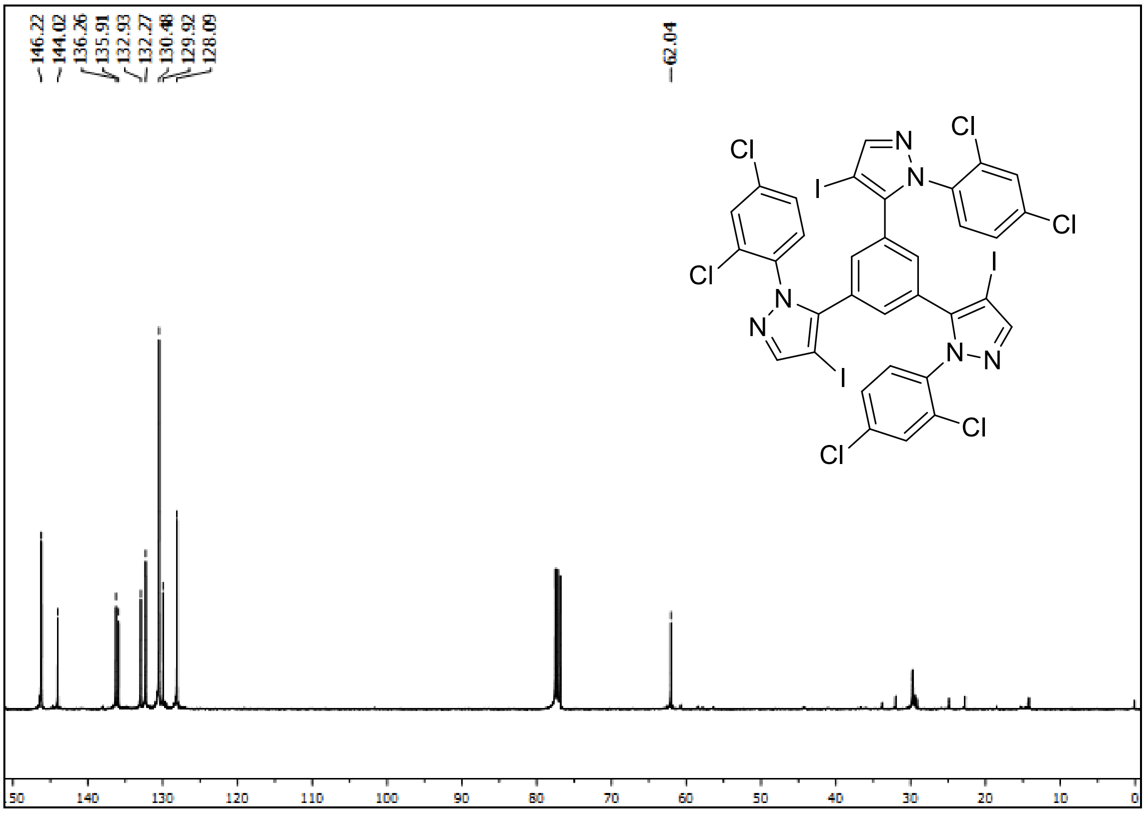


**Figure S26.** ¹³C NMR spectrum of 1,3,5-tris(4-iodo-1-(2,4-dichlorophenyl)-1H-pyrazol-5-yl)benzene **11c**.

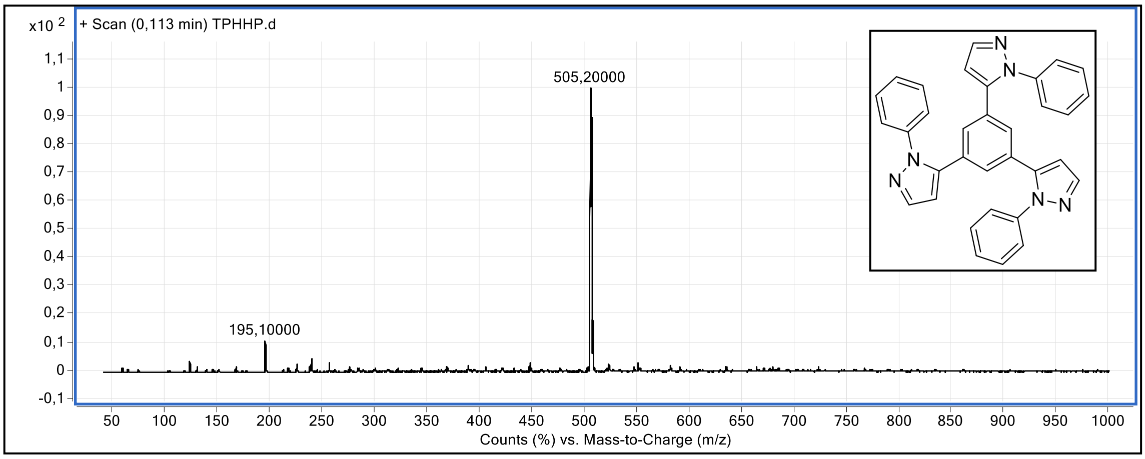


**Figure S27.** LCMS spectra NMR spectrum of 1,3,5-tris(1-phenyl-1H-pyrazol-5-yl)benzene **5**.

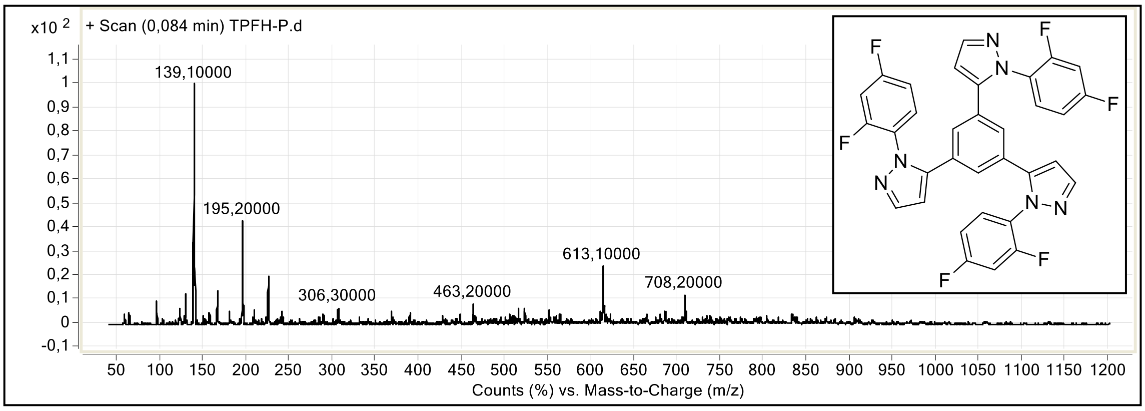


**Figure S28.** LCMS spectra of 1,3,5-tris(1-(2,4-difluorophenyl)-1H-pyrazol-5-yl)benzene **6**.

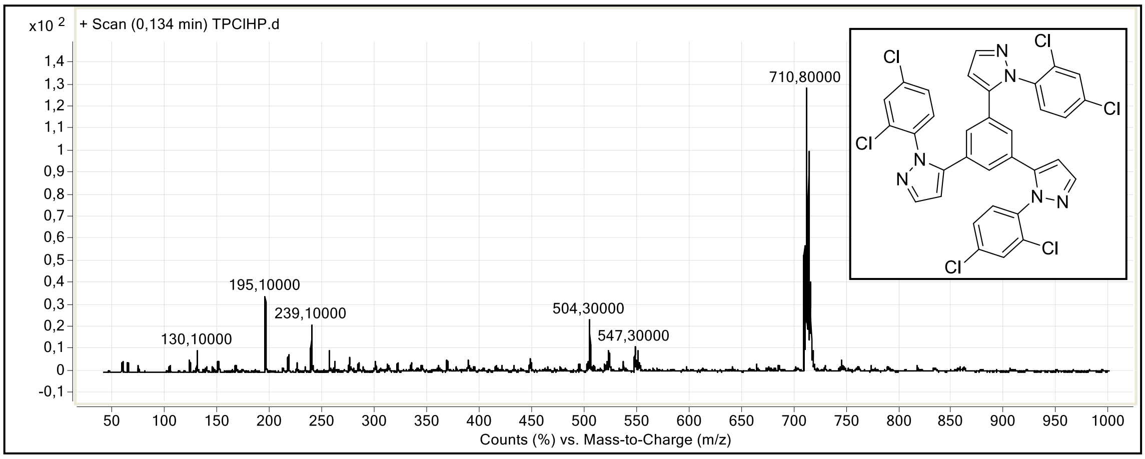


**Figure S29.** LCMS spectra of 1,3,5-tris(1-(2,4-dichlorophenyl)-1H-pyrazol-5-yl)benzene **7**.

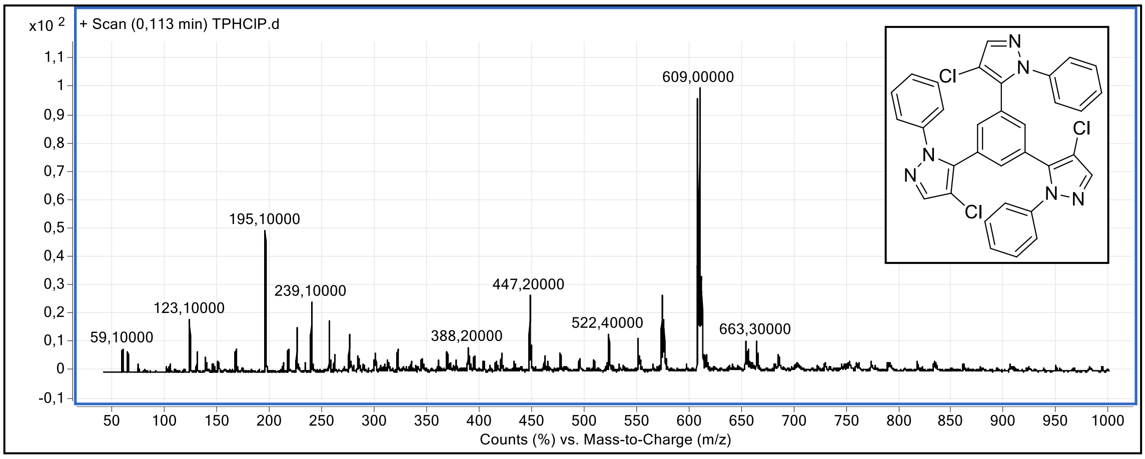


**Figure S30.** LCMS spectra of 1,3,5-tris(4-chloro-1-phenyl-1H-pyrazol-5-yl)benzene **9a**.

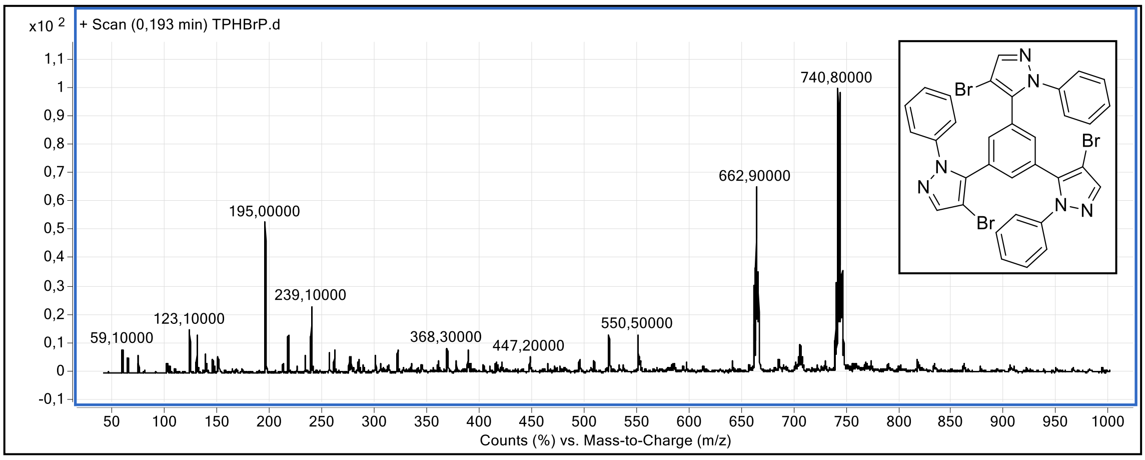


**Figure S31.** LCMS spectra of 1,3,5-tris(4-bromo-1-phenyl-1H-pyrazol-5-yl)benzene **9b**.

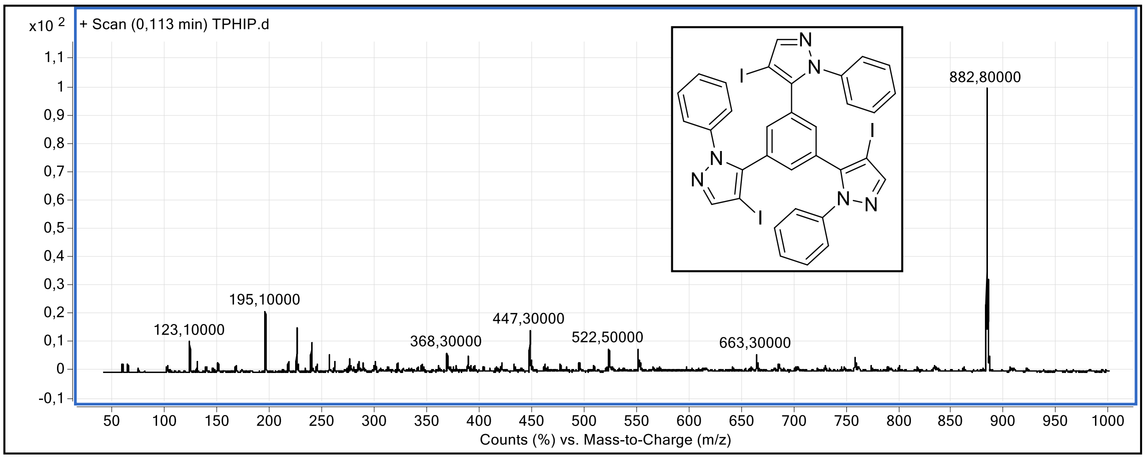


**Figure S32.** LCMS spectra of 1,3,5-tris(4-iodo-1-phenyl-1H-pyrazol-5-yl)benzene **9c**.

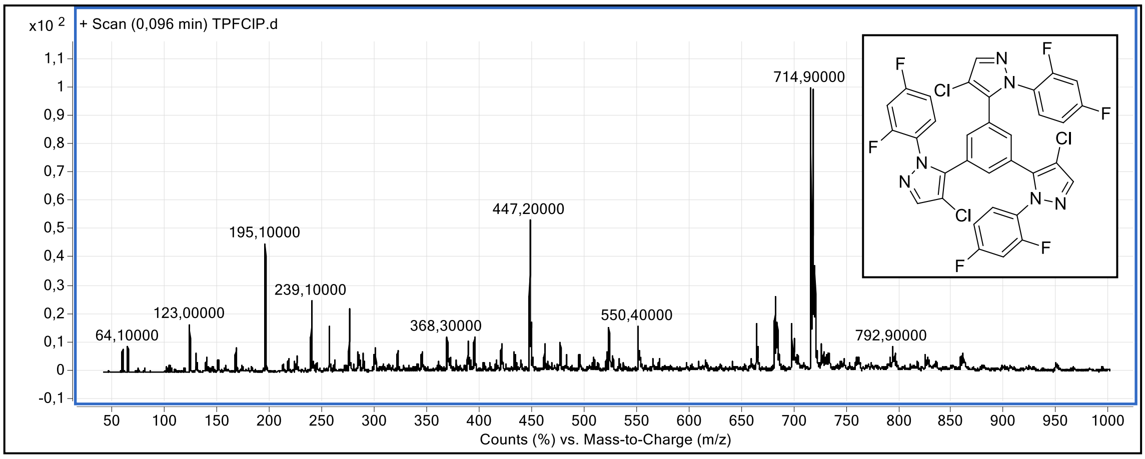


**Figure S33.** LCMS spectra of 1,3,5-tris(4-chloro-1-(2,4-difluorophenyl)-1H-pyrazol-5-yl)benzene **10a**.

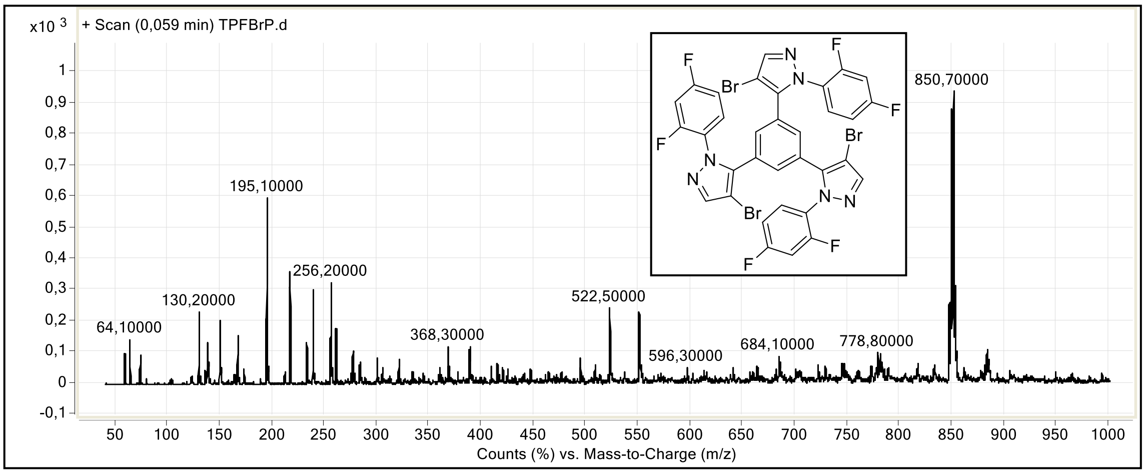


**Figure S34.** LCMS spectra of 1,3,5-tris(4-bromo-1-(2,4-difluorophenyl)-1H-pyrazol-5-yl)benzene **10b**.

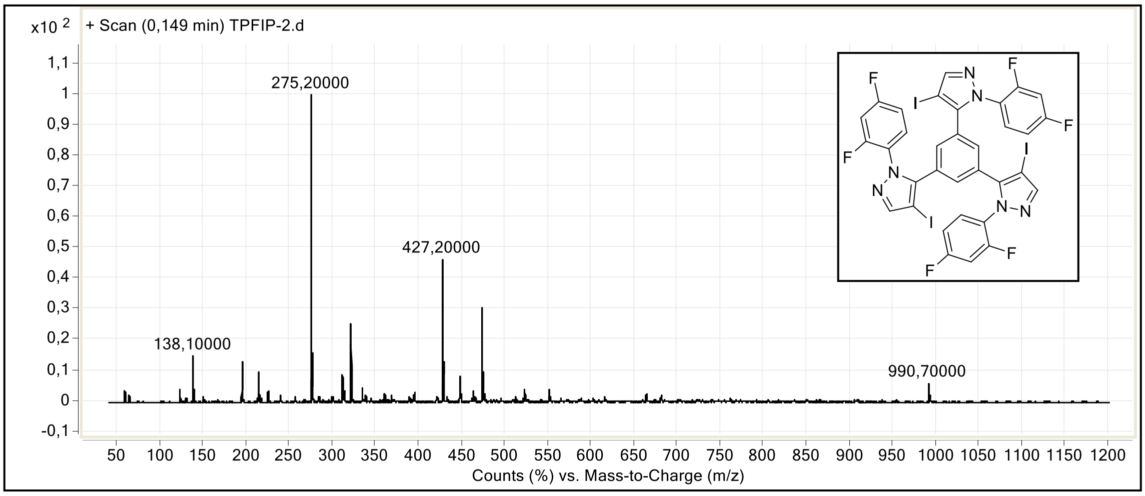


**Figure S35.** LCMS spectra of 1,3,5-tris(4-iodo-1-(2,4-difluorophenyl)-1H-pyrazol-5-yl)benzene **10c**.

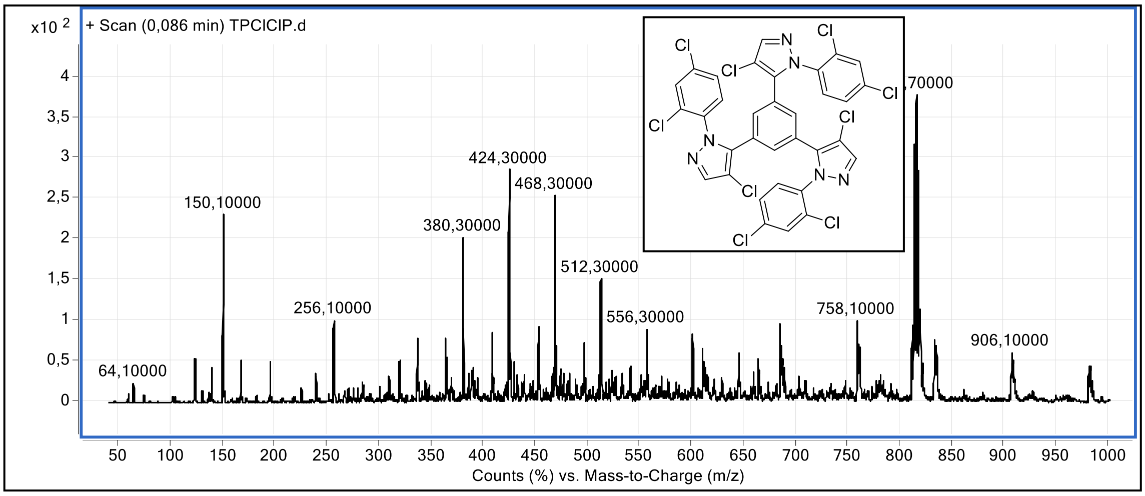


**Figure S36.** LCMS spectra of 1,3,5-tris(4-chloro-1-(2,4-dichlorophenyl)-1H-pyrazol-5-yl)benzene **11a**.

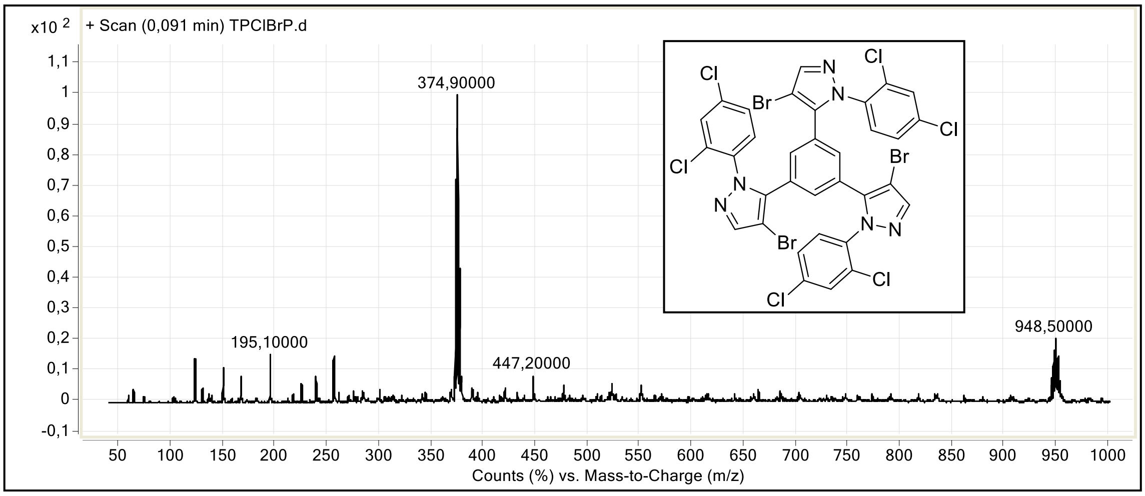


**Figure S37.** LCMS spectra of 1,3,5-tris(4-Bromo-1-(2,4-dichlorophenyl)-1H-pyrazol-5-yl)benzene **11b**.

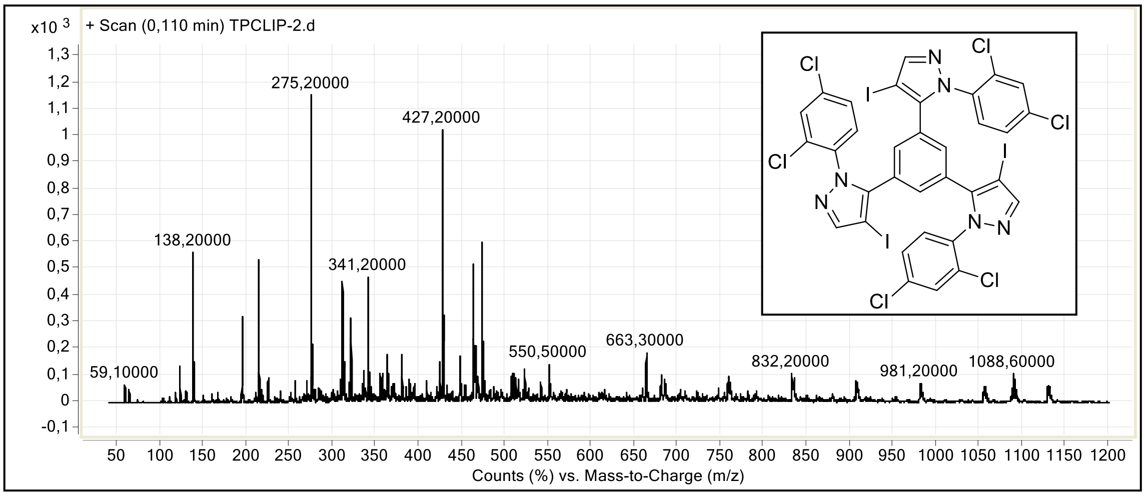


**Figure S38.** LCMS spectra of 1,3,5-tris(4-iodo-1-(2,4-dichlorophenyl)-1H-pyrazol-5-yl)benzene **11c**.
